# Recent advances in functionalized quinoline scaffolds and hybrids—Exceptional pharmacophore in therapeutic medicine

**DOI:** 10.3389/fchem.2022.1074331

**Published:** 2023-01-06

**Authors:** Oluwadunni F. Elebiju, Olayinka O. Ajani, Gbolahan O. Oduselu, Temitope A. Ogunnupebi, Ezekiel Adebiyi

**Affiliations:** ^1^ Covenant University Bioinformatics Research (CUBRe), Covenant University, Ota, Nigeria; ^2^ Department of Chemistry, College of Science and Technology, Covenant University, Ota, Nigeria; ^3^ Department of Computer and Information Science, Covenant University, Ota, Nigeria; ^4^ Division of Applied Bioinformatics, German Cancer Research Center (DKFZ), Heidelberg, Germany

**Keywords:** admet, drug design, hybrid, pharmacological activity, quinoline, synthesis

## Abstract

Quinoline is one of the most common nitrogen-containing heterocycles owing to its fascinating pharmacological properties and synthetic value in organic and pharmaceutical chemistry. Functionalization of this moiety at different positions has allowed for varying pharmacological activities of its derivative. Several publications over the last few decades have specified various methods of synthesis. This includes classical methods of synthesizing the primary quinoline derivatives and efficient methods that reduce reaction time with increased yield employing procedures that fulfill one of the twelve green chemistry principles, “safer solvent”. The metal nanoparticle-catalyzed reaction also serves as a potent and effective technique for the synthesis of quinoline with excellent atom efficiency. The primary focus of this review is to highlight the routes to synthesizing functionalized quinoline derivatives, including hybrids that have moieties with predetermined activities bound to the quinoline moiety which are of interest in synthesizing drug candidates with dual modes of action, overcoming toxicity, and resistance amongst others. This was achieved using updated literature, stating the biological activities and mechanisms through which these compounds administer relief. The ADMET studies and Structure-Activity Relationship (SAR) of novel derivatives were also highlighted to explore the drug-likeness of the quinoline-hybrids and the influence of substituent characteristics and position on the biological activity of the compounds.

## 1 Introduction

Heterocyclic compounds are cyclic compounds with atoms different from carbon within the ring. These atoms are referred to as heteroatoms, they include oxygen, nitrogen, sulfur, *etc.* The presence of heteroatoms allows for functionalities and activities, making them of critical importance for medicinal chemists (Hossain, 2018). The most prominent bioactive chemical compounds are nitrogen-containing heterocycles, which are found in natural products (as alkaloids in a range of plants), physiologically active synthetic chemicals, medications, and other products (Teja and Khan, 2020).

**FIGURE 1:**
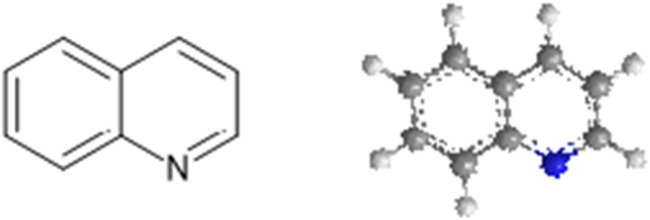
Chemical and 3D structure of quinoline.

**FIGURE 2:**
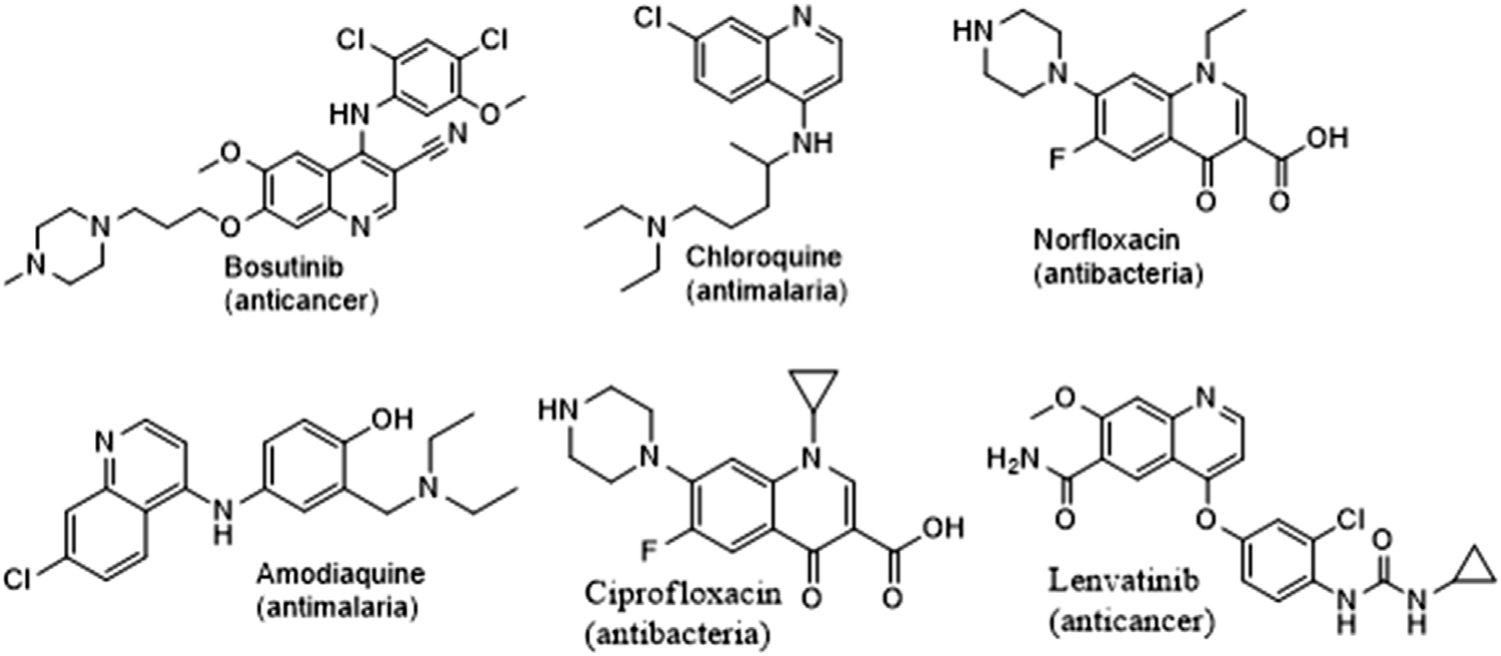
Structures of some commercially available quinoline containing drugs.

Quinoline is a nitrogen-containing fused bicyclic heterocycle with the chemical formula C_9_H_7_N (Figure 1). It is also known as benzo [*b*]pyridine. Its derivatives have been used in many fields, including medicine (Desai et al., 2021). Figure 2 shows some commercially available drugs with quinoline moiety. Quinoline is a multifunctional scaffold in medicinal chemistry that forms a salt with acids and undergoes electrophilic and nucleophilic substitution reactions (Yadav and Shah, 2021). This property allows for functionalization at numerous ring positions and its use as an intriguing synthetic building block in the design of drugs and their synthesis. The substituent characteristics and position are important to note as they play a significant role in the functionality of synthesized compounds, allowing for a wide range of applications and biological activities (Abdelbaset et al., 2018).

**FIGURE 3:**
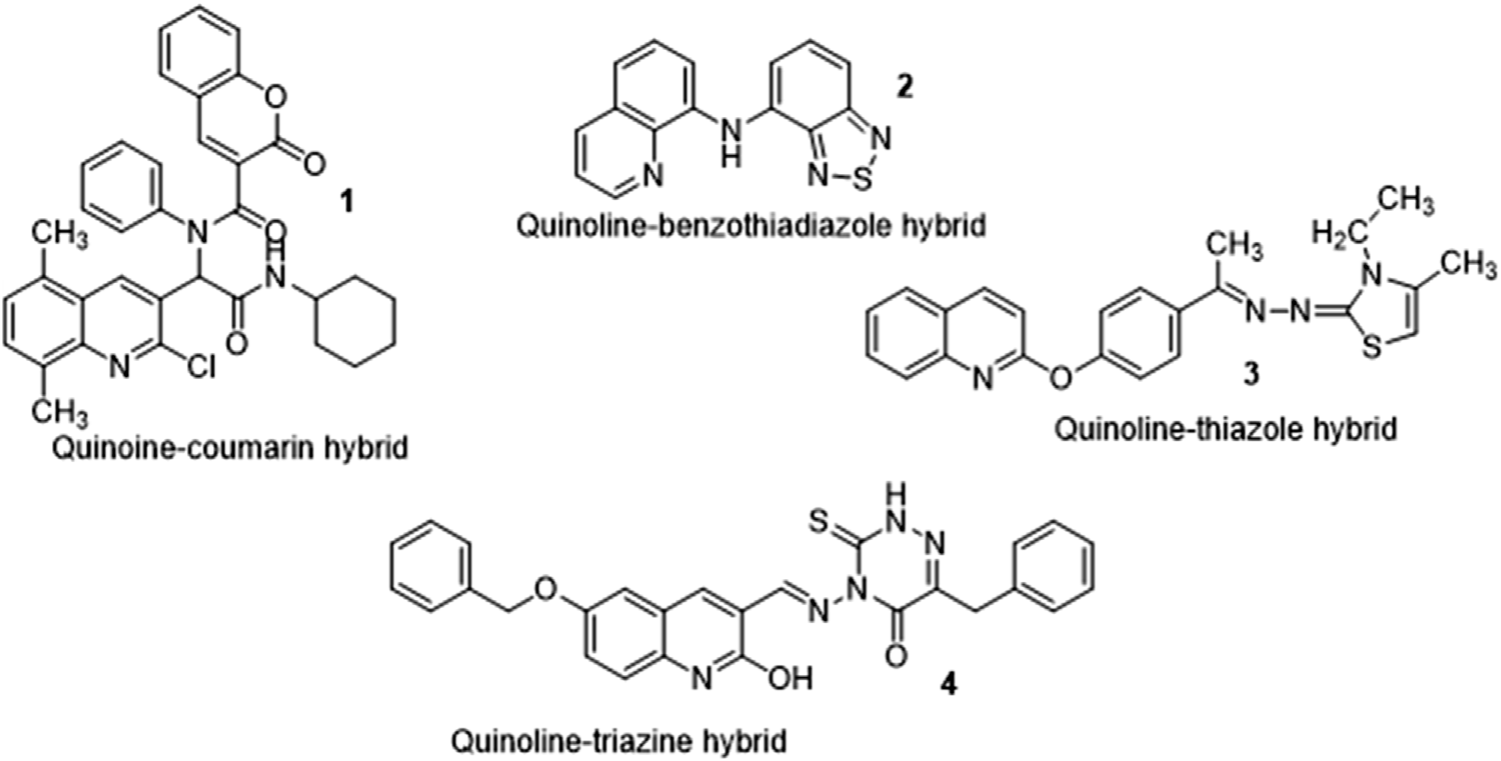
Structures of reported quinoline hybrids.

Quinoline and its derivatives have been of interest to scientists, with many chemists seeking to find more effective synthetic methods by improving on existing synthesis *via* reaction optimization (Yadav and Shah, 2021). The growing resistance of disease-causing organisms to commercially available drugs and increasing toxicity exhibited as side effects have allowed for the synthesis of potential drug candidates involving a combination of two biologically active molecules into one single hybrid entity. Quinoline hybrids have been synthesized in recent years and reported to have improved activity when compared to standard drugs used. Examples shown in Figure 3 are the Quinoline-coumarin hybrid (Taheri et al., 2019), the Quinoline-benzothiadiazole hybrid (Medeiros et al., 2021), the Quinoline-thiazole hybrid (Eissa et al., 2021), Quinoline-triazine hybrid (Ghanim et al., 2022), amongst others. This review focuses on the synthesis, pharmacological activity, Structure-Activity Relationship (SAR), and ADMET studies of quinoline derivatives including quinoline hybrids to uncover their drug-likeness and the activities responsible for the novel pharmacological activity.

## 2 Chemistry of quinoline

### 2.1 Synthesis of quinoline derivatives

Quinoline and its various derivatives have been demonstrated to be quite easy to produce by, utilizing various synthetic approaches to obtain pharmacologically active derivatives (Yadav and Shah, 2021). Some of the synthesis techniques are.

#### 2.1.1 Classical synthesis of quinoline

**FIGURE 4:**
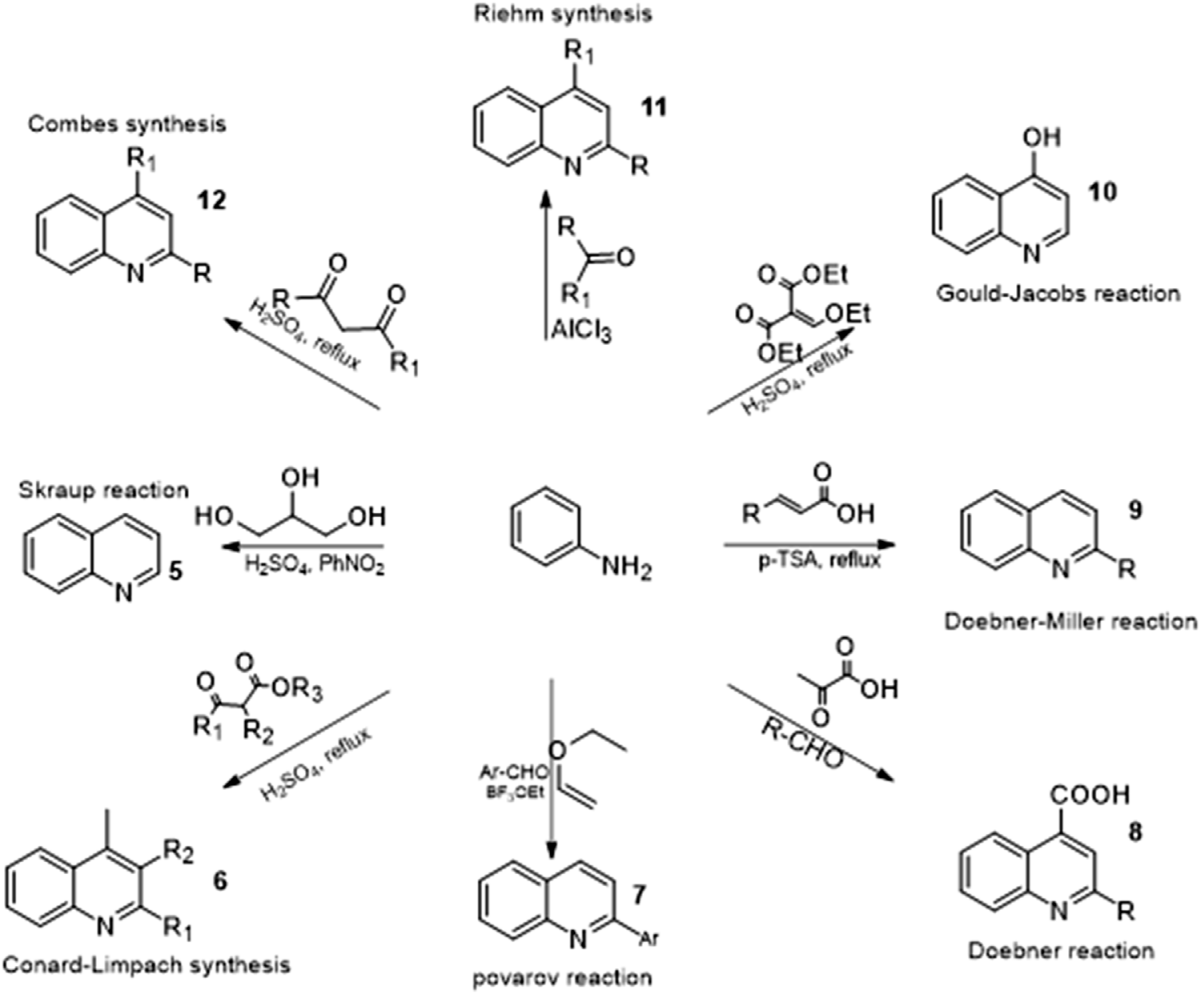
Classical synthetic route of quinoline.

The quinoline motif can be synthesized using a variety of classical synthetic methods. These classical methods have been employed in recent years in the synthesis of functionalized quinoline derivatives. Popular synthetic pathways that include aniline as reactants are shown in Figure 4. Substituted aniline is also used as a reactant to achieve derivatives that can be further functionalized at strategic positions (Nainwal et al., 2019).

**SCHEME 1:**
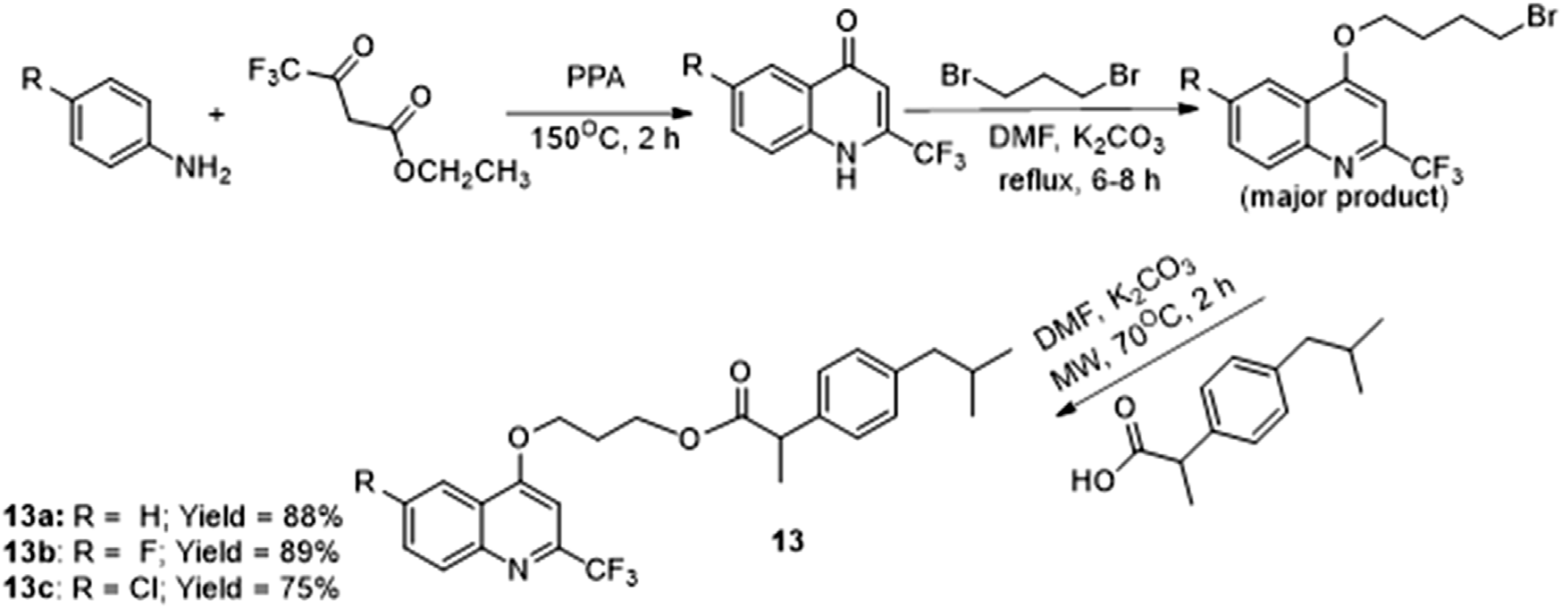
Conrad-Limpach cyclo condensation for the synthesis of ibuprofen-quinoline hybrid.

Ghanim et al. used the Conrad-Limpach cyclo condensation approach as in (Scheme 1) to react *p*-substituted aniline and ethyl 4,4,4-trifluoro-3-oxobutanoate to synthesize a di-substituted quinolone which was an intermediate in the preparation of ibuprofen-quinoline hybrids. Compounds **13a, 13b, and 13c** had the most promising anti-inflammatory activities. The color of the hybrids ranged from colorless oil to white microcrystals and the melting points of the solid crystals fell between (60–70)^o^C (Ghanim et al., 2022).

**SCHEME 2:**
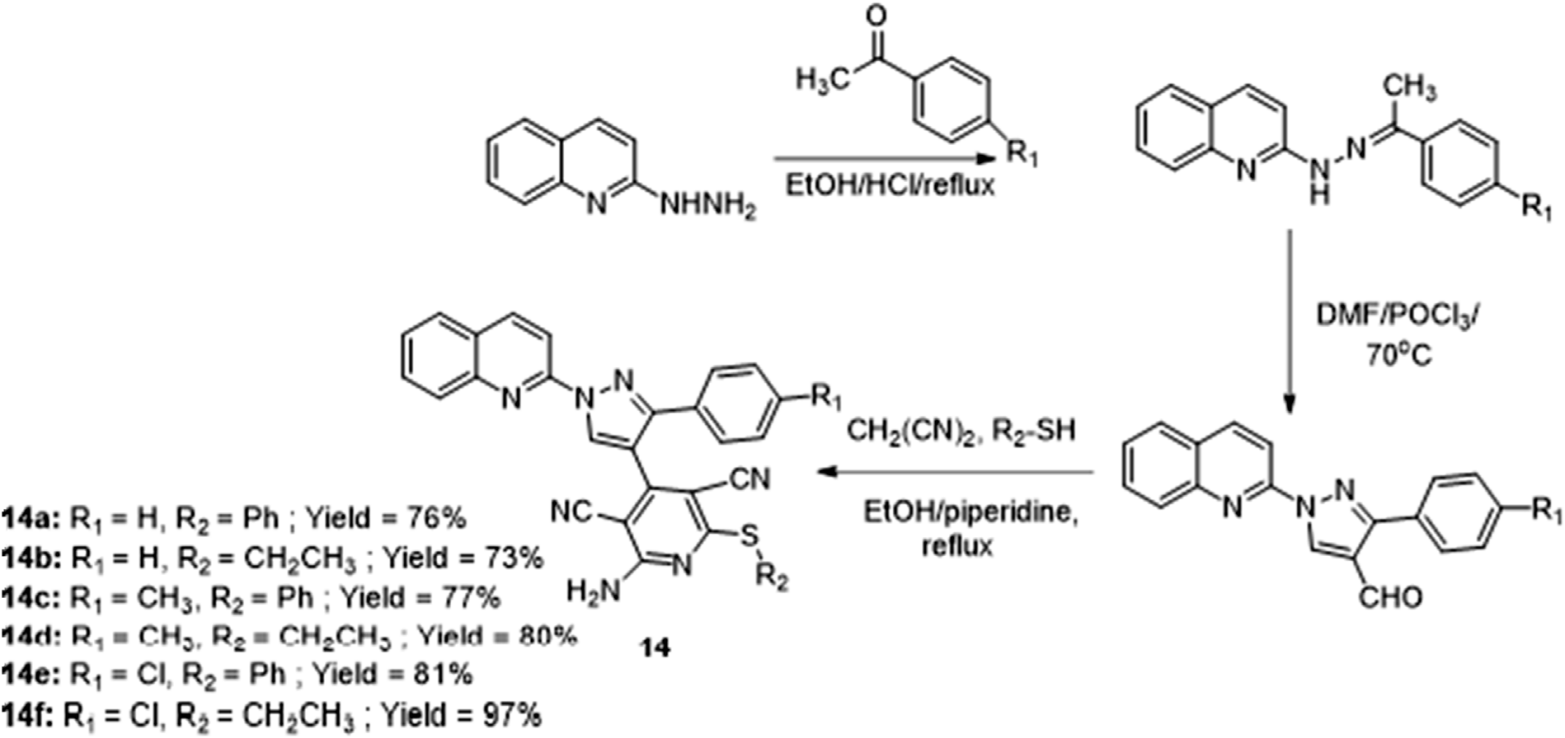
Vilsmeier-Haack synthesis of pyrazole and pyridine-quinoline hybrids.

Amer et al. employed the Vilsmeier-Haack procedure to synthesize pyrazole-pyridine-based quinoline hybrids (Scheme 2), *via* the formylation of quinoline hydrazones to form the 4-formyl pyrazole derivatives. In a one-pot synthesis, 4-formyl pyrazole derivatives were combined with malononitrile and thiophenol to produce 2-substituted quinoline derivatives. All derivatives **14a-f** were reported to exhibit broader antimicrobial activities when compared to standards. The hybrids were yellow in color with melting points between (160–200)^o^C and others greater than 250°C (Mahgoub, 2018).

**SCHEME 3:**
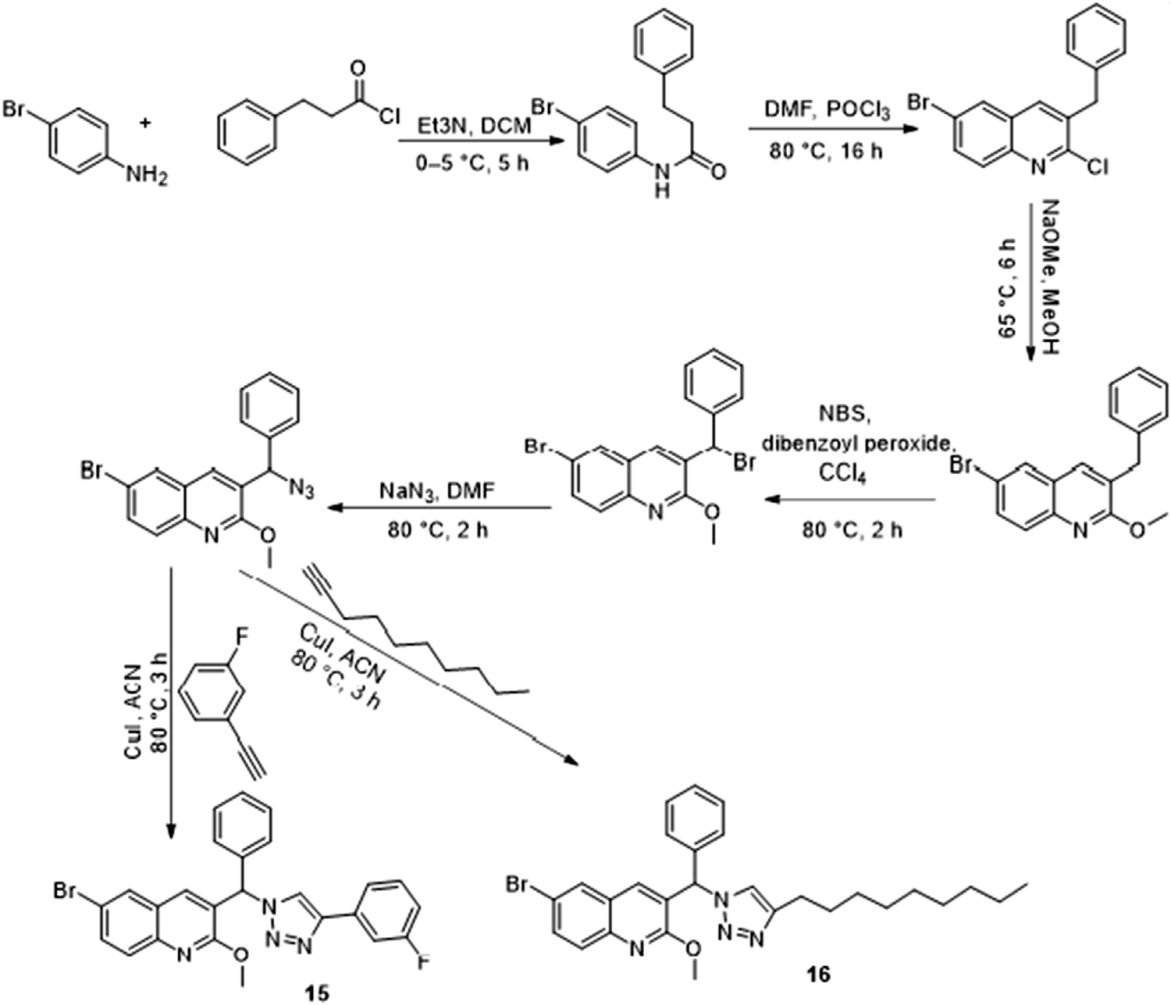
Vilsmeier-Haack synthesis of quinoline-triazole hybrids.

Ramprasad et al. (Scheme 3), reacted bromo-substituted aniline with phenylpropanoid chloride and triethylamine in DCM, which was formylated and subsequently cyclized through the Vilsmeier–Haack reaction. This was followed by a substitution reaction of the chloro group with a methoxy group. The intermediate was then treated with n-bromosuccinamide (NBS), and tetrachloromethane (CCl_4_) to give a derivative that was then transformed into a substituted azide compound. This intermediate was then reacted with substituted alkynes in acetonitrile under reflux conditions to give the corresponding quinoline-triazole hybrids with melting points ranging from (73–220)^o^C. Derivatives including compounds 15 and 16 which were reported to be the most active among the series against *Mycobacterium bovis* (Ramprasad et al., 2019).

**SCHEME 4:**
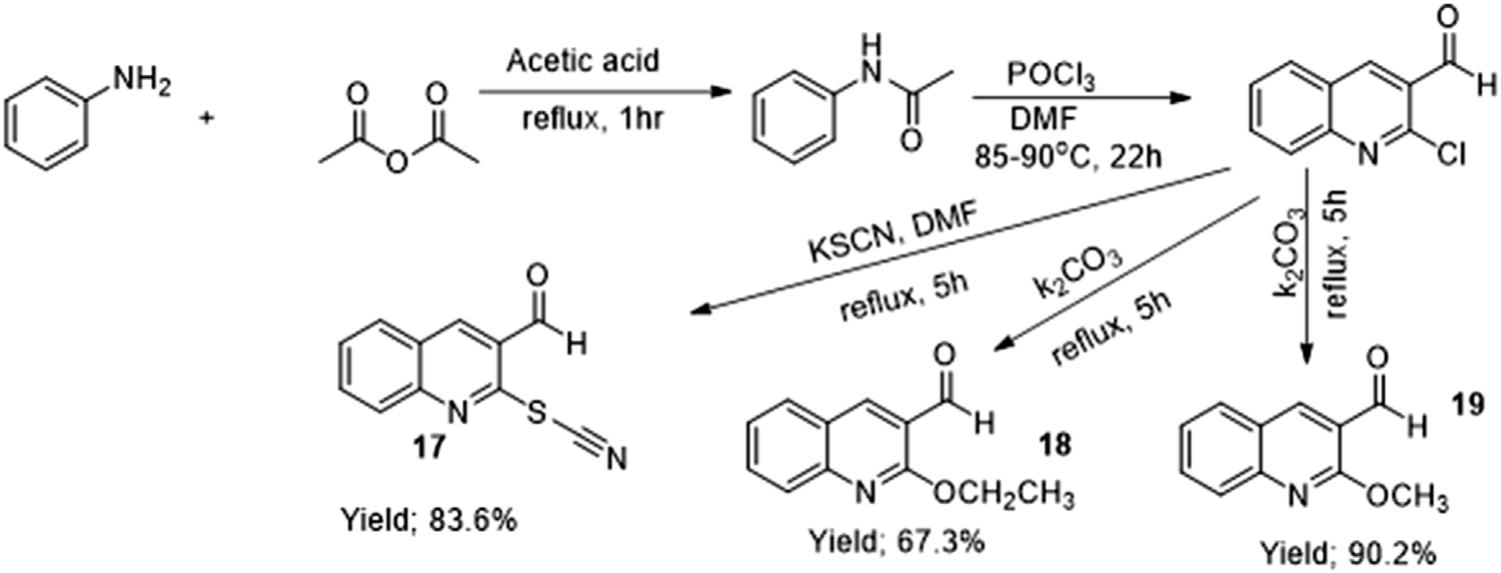
Vilsmeier-Haack synthesis of quinoline-carbaldehyde derivatives.

Zeleke et al. used the Vilsmeier-Haack reaction to synthesize quinoline-carbaldehyde derivatives (Scheme 4) from acetamide derivatives which were previously prepared by acetylation of aniline. Under different reaction conditions, substitution *via* an aromatic nucleophilic approach was done to introduce alternative nucleophiles resulting in the formation of new derivatives. Compounds **17**,**18** and **19** were some of the reported derivatives. The color of derivatives ranged from white to grey, to orange and yellow, with melting points between 90–180) ^o^C (Zeleke et al., 2020).

#### 2.1.2 Microwave-assisted synthesis

Environmental concerns have risen in chemical research, prompting the development of a variety of concepts to eliminate and reduce waste production. A microwave uses dipole rotation and ionic conduction to transport energy directly to reactive species. Microwaves generate electric and magnetic fields, but the electric field alone is used to heat a material, allowing a cut down on both the amount of energy used and the time it takes for a reaction to happen (Nainwal et al., 2019). It is an environmentally friendly reaction as it takes place in enclosed chambers.

**SCHEME 5:**
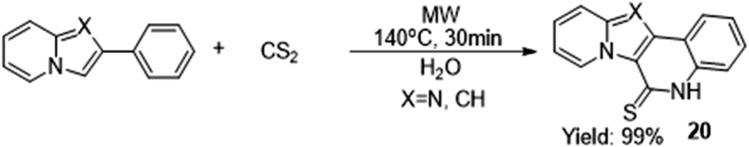
Microwave-assisted synthesis of fused quinoline-thiones.

Li et al. synthesized derivatives of quinoline-thiones fused with poly-heterocyclic by reacting pyridine-imidazole derivative with carbon disulfide in different bases *via* a microwave annulation process through 6πelectrocyclization to create scaffolds. The solvent used and time were varied while the temperature was kept constant. Reaction conditions reported in Scheme 5 gave the best yield of 99% using water as solvent. It was reported that microwave-assisted heating generally improved reaction efficiency (Li et al., 2020).

**SCHEME 6:**
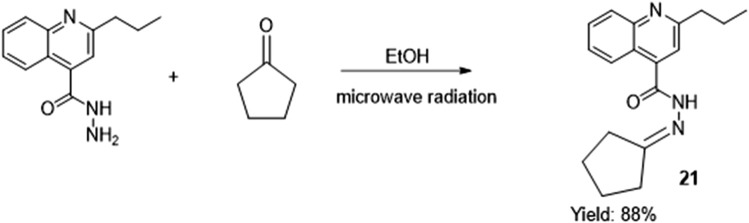
Microwave-assisted condensation of hydrazide-hydrazone quinoline derivatives.

Ajani et al. synthesized hydrazide-hydrazone derivatives (Scheme 6) *via* microwave-assisted condensation reaction of the NH_2_ free end of carbohydrazide derivative with various aliphatic and alicyclic ketones carbonyl centers that are sp^2^ hybridized in ethanol. The reaction took between 1 and 3 min to complete due to its exposure to microwave radiation, and the melting points of most of the derivatives were greater than 300°C. Compound **21** was one of the reported compounds (Ajani et al., 2018).

#### 2.1.3 Ultrasound-assisted synthesis

Ultrasound-assisted multicomponent reactions in water are good instruments for generating bioactive chemicals. This is because they provide higher yields than other approaches. They have been used in the synthesis of a range of heterocyclic compounds.

**SCHEME 7:**
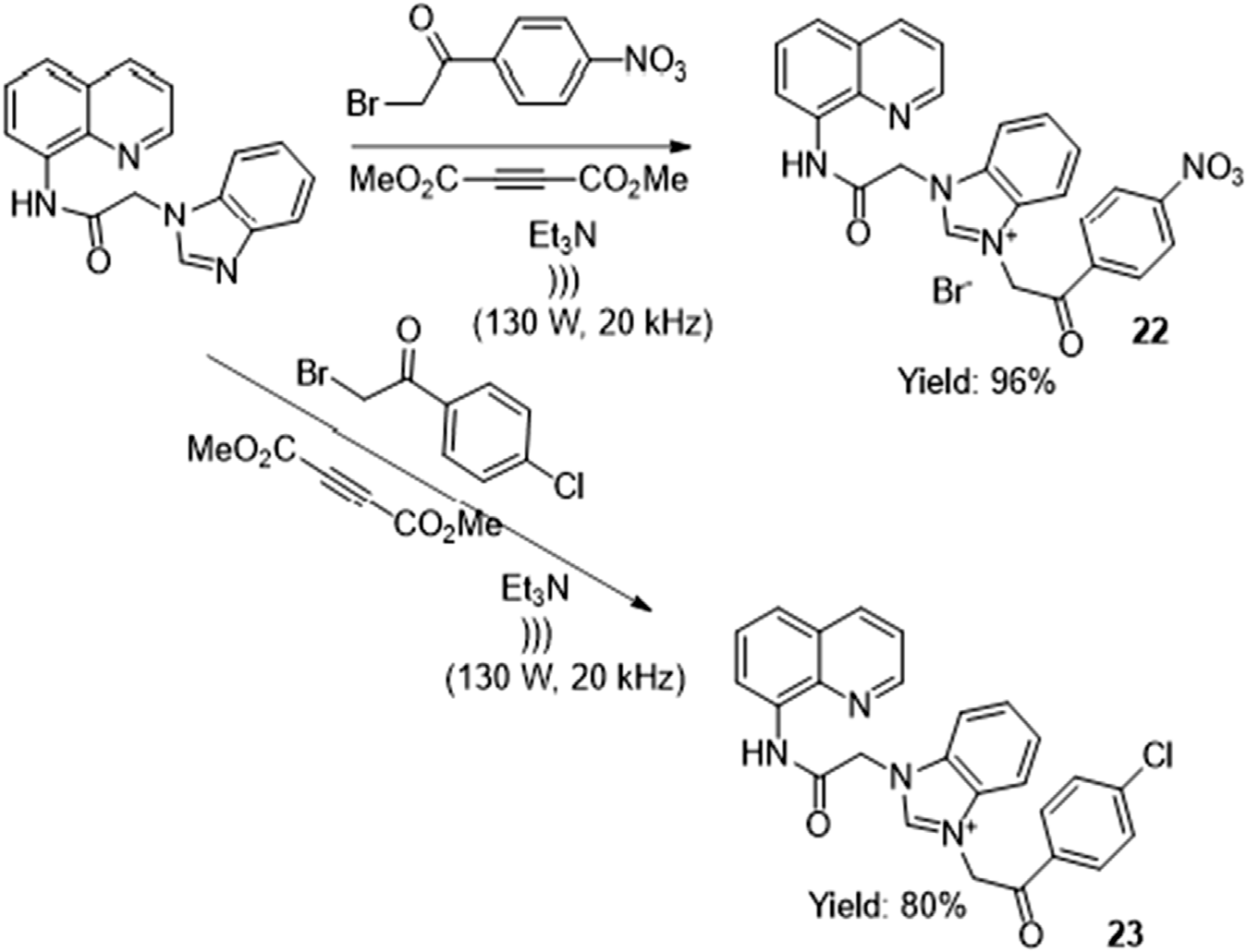
Ultrasound-assisted quinoline imidazolium salts synthesis.

Diaconu et al. used ultrasound irradiation to produce the quinoline-imidazolium hybrids by *N*-alkylation of the acidic nitrogen in benzimidazole with substituted ω-halogen acetophenones (Scheme 7). All derivatives including compounds **22** and **23** were reported to have improved yield when compared with conventional heating methods (Diaconu et al., 2021).

**SCHEME 8:**
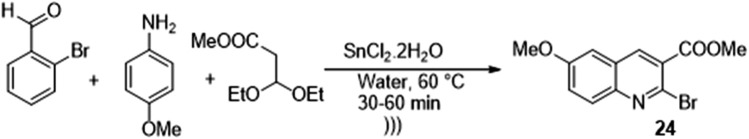
Ultrasound-assisted synthesis of 2-substituted quinolones.

Upadhyay et al. synthesized and investigated 2-substituted quinoline derivatives (Scheme 8), one of which is **24** by one-pot three-component fusion of methyl 3,3-diethoxypropionate, varying aniline derivatives, and aldehyde at 60°C under ultrasonic circumstances utilizing SnCl_2._2H_2_O as a catalyst and water as solvent (Upadhyay et al., 2018).

#### 2.1.4 Transition metal nanoparticles mediated synthesis

**SCHEME 9:**
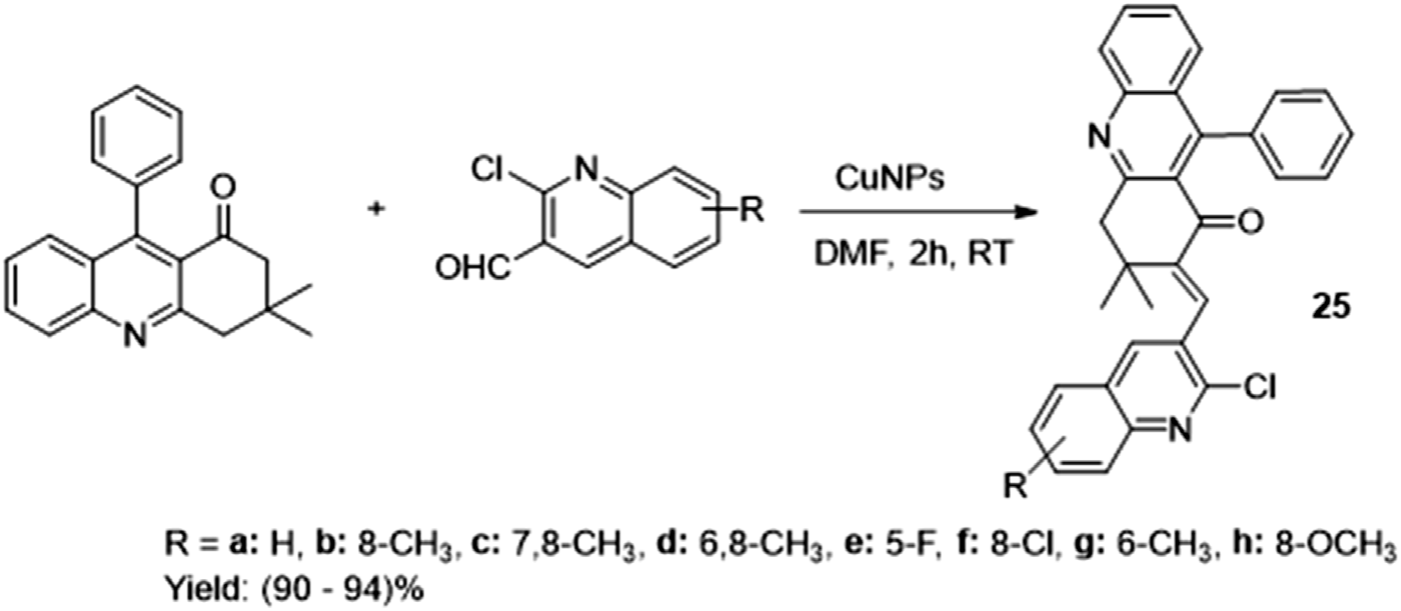
CuNPs mediated Knoevenagel condensation.

Angajala et al. used the Knoevenagel condensation of quinoline-carbaldehyde and cyclohexanone-fuse derivatives to produce quinoline-acridine hybrids (Scheme 9). They used copper nanoparticles as a catalyst, stirring for 2 h at room temperature in DMF. Compound **25** represents the hybrids where the position and identity of ‘R’ are dependent on the carbaldehyde derivative used. The synthesized derivatives were confirmed using NMR (^1^H and ^13^C) which confirmed the number of proton and carbon atoms with their respective chemical shift (Angajala et al., 2020).

#### 2.1.5 Click chemistry-supported synthesis

**SCHEME 10:**
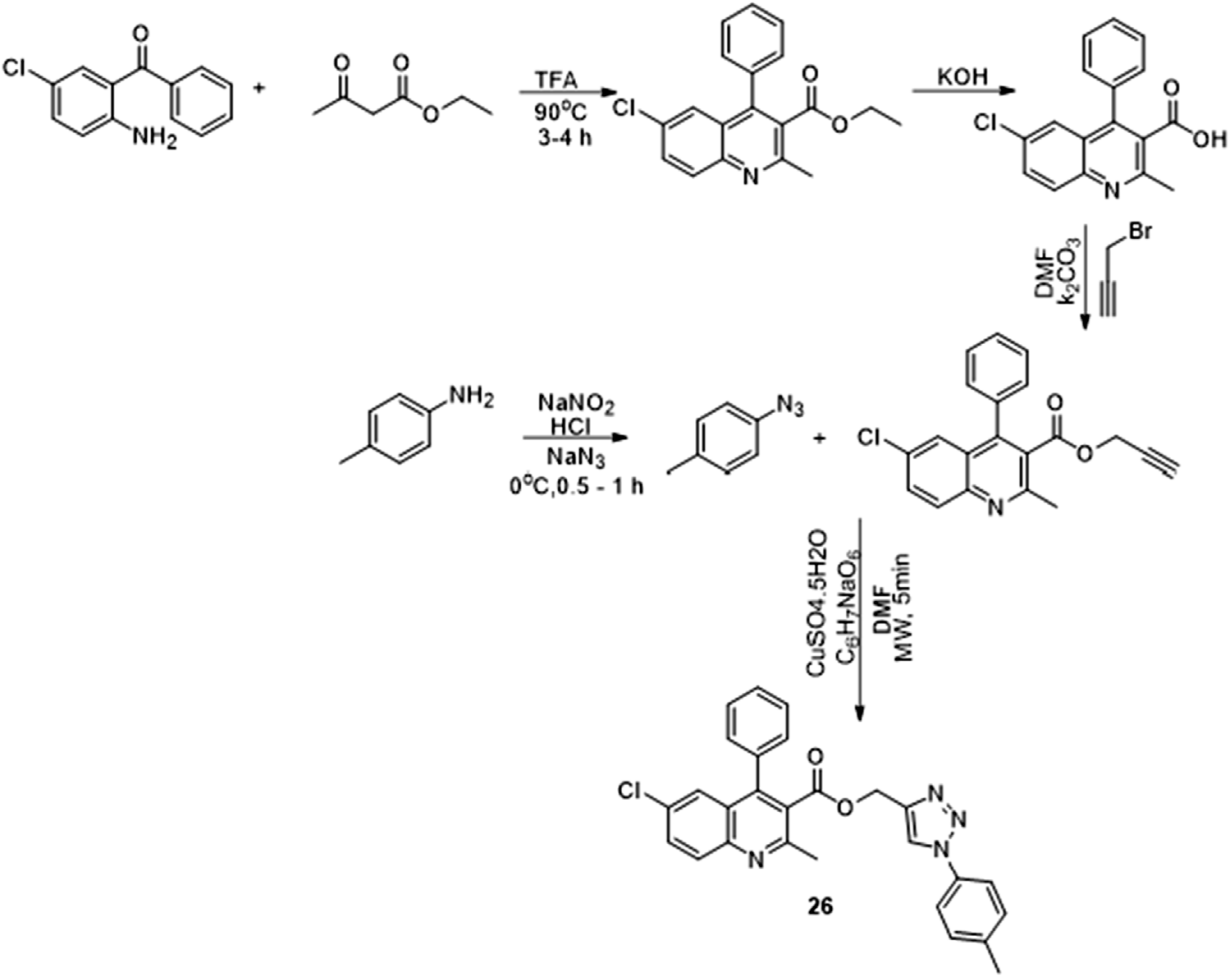
Synthesis of quinoline hybrid *via* click chemistry.

Upadhyay et al. designed and synthesized a series of quinoline derivatives using molecular hybridization approach supported by click chemistry (Scheme 10). Most of the derivatives were white solids with melting points above 100°C, compound **26** was one of the derivatives (Upadhyay et al., 2018).

## 3 Activities of some quinoline motifs

### 3.1 Pharmacological activities

In a bid to investigate their pharmacological activities, reacting quinoline scaffolds with moieties having pharmacophore characteristics have been explored. This has led to the production of hybrids, some of which have been reported to possess higher pharmacological activity when compared to commercially available quinoline-based medicines indicating a synergistic effect of moieties introduced.

#### 3.1.1 Antimicrobial activities

Amer et al. *via in vitro* analysis tested pyrazole- and pyridine-based quinoline hybrids for both antibacterial and antifungal activities. Antifungal analysis was conducted against *Candida albicans* using Ketoconazole as standard at a concentration of 100 μg/mL while antibacterial screening against two Gram-positive bacteria (*Staphylococcus aureus* and *Bacillus subtilis*), and two Gram-negative bacteria (*Salmonella typhimurium* and *Escherichia coli*) was carried out using Ciprofloxacin as standard. Some of the synthesized derivatives, such as **15**, displayed far broader antibacterial activity than Ciprofloxacin, while others demonstrated good to moderate antimicrobial performance against the pathogens examined. These compounds were found to have antibacterial activity against a variety of bacteria (Mahgoub, 2018).

**FIGURE 5:**
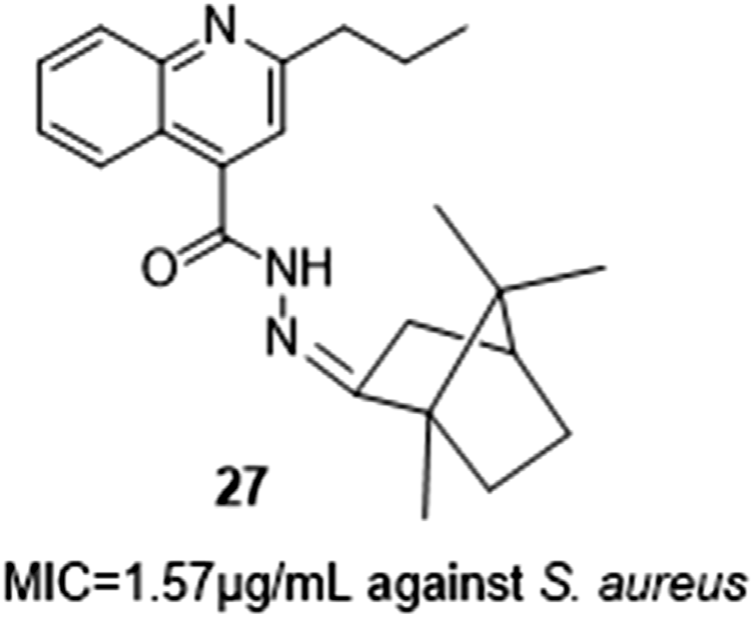
Hydrazide-hydrazone-quinoline hybrids with antimicrobial activity.

Ajani et al. synthesized a series of hydrazide-hydrazone-quinoline hybrids using microwave irradiation (Figure 5). When compared to gentamicin, most of the novel quinoline hydrazide-hydrazones were reported to have higher inhibitory power, the most active being **27** having MIC values from 1.59 to .39 μg/mL against *Staphylococcus aureus*, *Bacillus lichenformis*, *Micrococcus varians, Escherichia coli,* and *Proteus vulgar* (Ajani et al., 2018).

#### 3.1.2 Antitubercular activities

**FIGURE 6:**
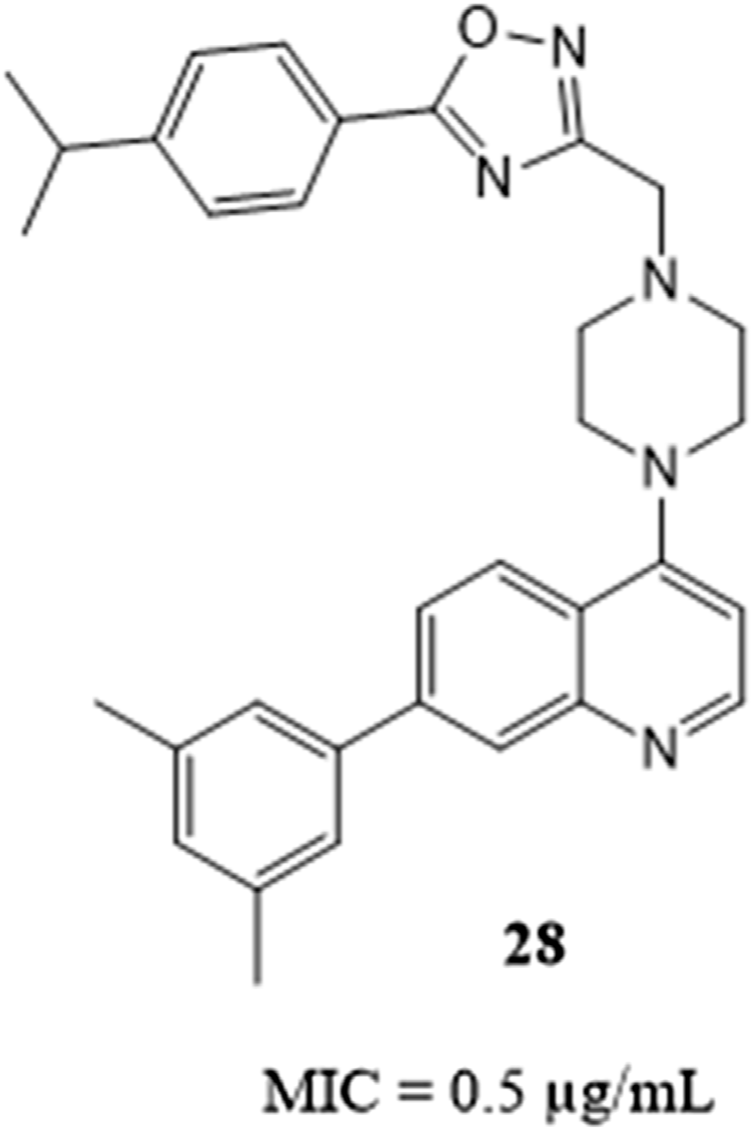
Quinoline-oxadiazole hybrid with antitubercular Activities.

Multidrug-resistant tuberculosis (MDR–TB) has been a significant obstacle in the fight against tuberculosis around the world. The quinoline-oxadiazole hybrids were synthesized by Shruthi et al. as a novel family of TB-specific compounds. Compound **28** with a MIC value of .5 μg/mL was reported to have the highest activity against Mtb WT H_37_Rv (Figure 6). Pharmacokinetics (PK) studies demonstrated that it is orally bioavailable having blood levels above the MIC of 2.5 μg/mL. The compounds produced were found to be metabolically stable, bioavailable, non-toxic, and exhibited good PK values, making them suitable for further research in a TB infection on an animal model (Shruthi et al., 2020).

Ramprasad et al. developed target quinoline-triazole hybrids and evaluated them against the growth of *Mycobacterium bovis*. The test compounds strongly reduced *Mycobacterium bovis* growth in antitubercular screening tests, with two of the derivatives **15** and **16**, having MIC values of 31.5 and 34.8 µM respectively. The findings of the compounds that demonstrate promising activity stress the importance of future research and the development of new antitubercular medications (Ramprasad et al., 2019).

#### 3.1.3 Antiproliferative activity

Nitric oxide release and induction of apoptosis are some of the mechanisms nitrones and oximes use to exhibit antiproliferative properties. Activation of caspase (a group of key apoptosis mediators) occurs when reactive nitrogen species (RNS) including NO_2_ and N_2_O_3_ targets P53. Caspase-3 which is the most important one can activate death protease and cleave some important cellular proteins (Porter and Jänicke, 1999).

**FIGURE 7:**
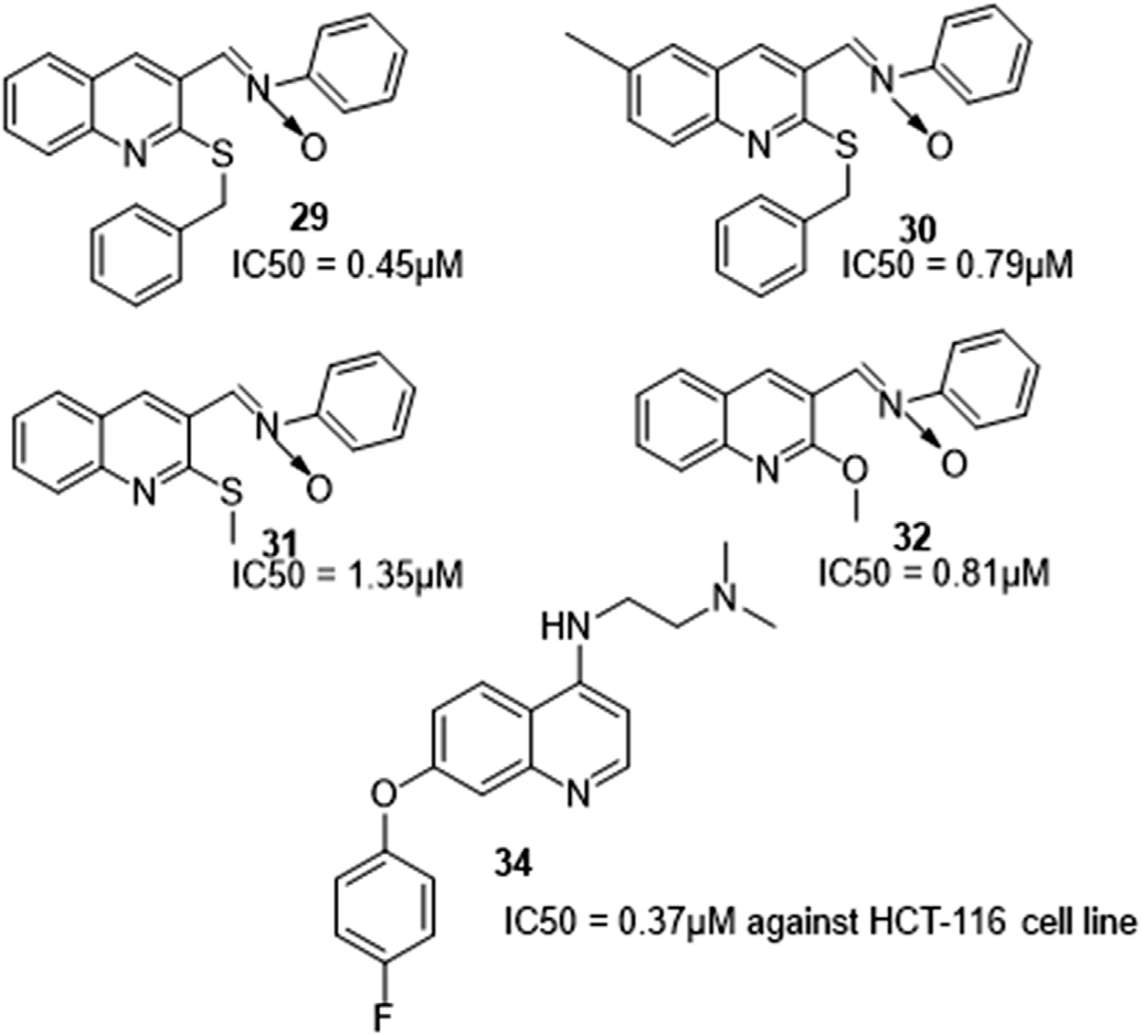
Quinolone derivatives with antiproliferative activity.

Abdelbaset et al. synthesized quinolone nitrones and quinoline oxime derivatives and determined the NO release of derivatives. They reported that nitrones release more NO than oximes on average and this contributed to them exhibiting increased antiproliferative activity than the oxime derivatives. Compounds **29, 30, 31**, and **32** which are quinoline-nitrones derivatives had a high percentage of inhibition of growth and antiproliferative action as opposed to the quinoline oximes derivatives which were ineffective (Figure 7). The nitrone derivatives with IC_50_ values ranging from .45–.91 µM against RPMI-8226 leukemia cell line were found to be more potent than the standard medicine doxorubicin. IC_50_ values ranging from .98–2.98 µM was recorded against HCT-116 colon cancer cell lines which were equivalent to that of the standard drug. A considerable rise in the level of caspase-3 protein in RPMI-8226 was observed when derivatives were evaluated as caspase-3 activators (Abdelbaset et al., 2018).

Quinoline derivatives with enhanced water solubility and antiproliferative action were synthesized by Li et al. *via* the introduction of a flexible alkylamino side chain at position-4 and an alkoxy group at position-7 of the quinoline nucleus. The derivatives were evaluated as potential antiproliferative drugs based on the chemical structure of the lead derivative. Preliminary SAR analysis suggested that big and bulky substituents at position-7 facilitated antiproliferative activity and the presence of amino side-chain substituents enhanced this activity. The length of the alkylamino side chain moiety also influenced antiproliferative potency, especially for derivatives with two CH_2_ units. Compound **34** was found to be the most effective among the derivatives (Li et al., 2019).

#### 3.1.4 Antileishmanial activity

**FIGURE 8:**
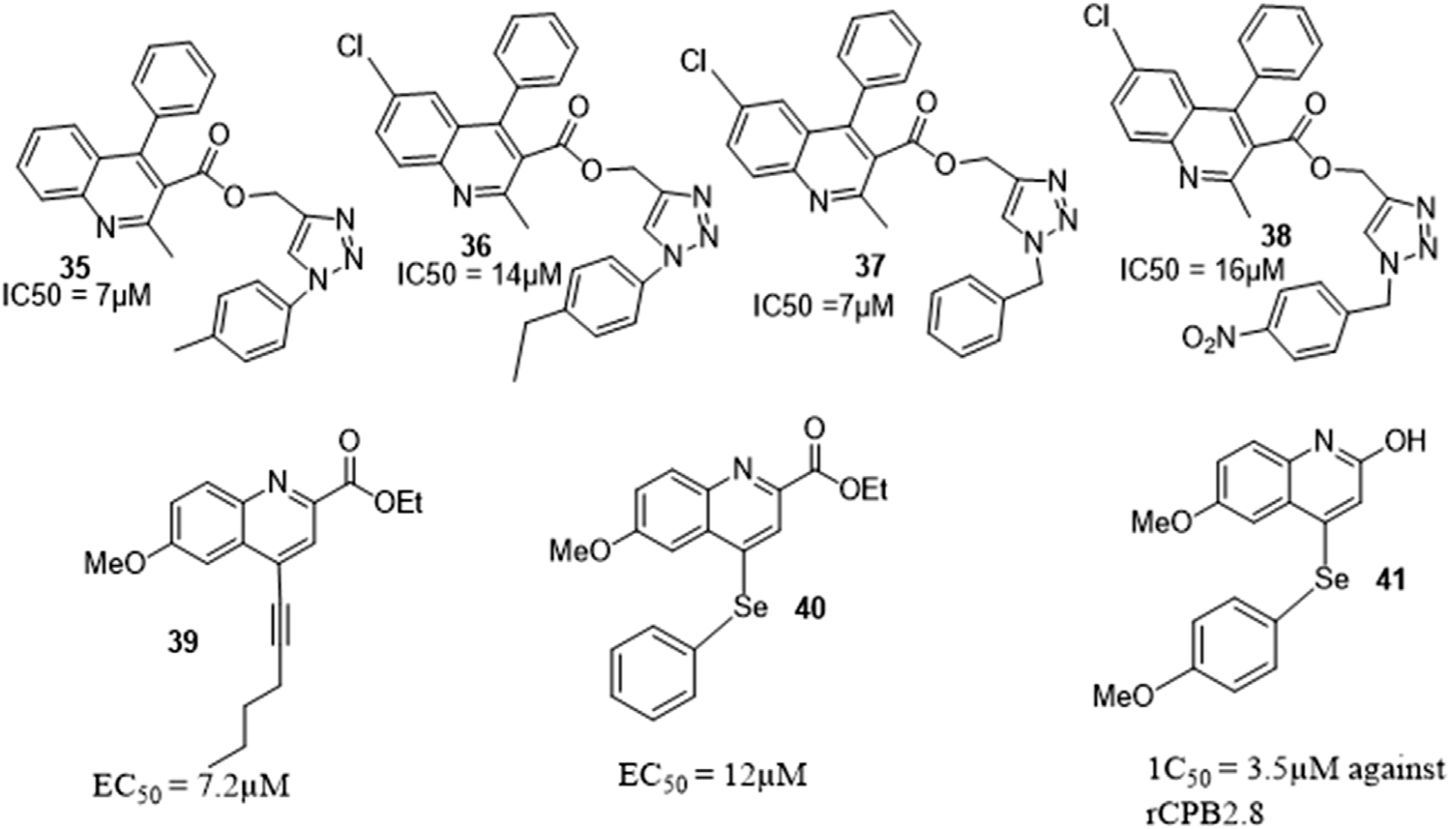
Quinoline hybrids with antileishmanial activity.

Macrophages play an important role in the cellular immune response and are activated as the first line of defense by host against Leishmania *sp*. Activation lead to increase in intracellular calcium levels and nitric oxide production which plays a role in parasite death (Muálem de Moraes Alves et al., 2021). Upadhyay et al. discovered that imidazo-quinoline hybrids *via* macrophage activation exhibited antileishmanial activity, hence synthesized series of quinoline-triazole hybrids which were evaluated as possible antileishmanial agents on cutaneous leishmaniasis as adjunct to antimonial using experimental models and clinical studies (Figure 8). Among all the derivatives, therapeutic *in vitro* action was observed for compounds **35, 36, 37**, and **38** against intracellular amastigotes of *Leishmania donovani*. Using the golden hamster model, *in vivo* antileishmanial activity of *L. donovani* was investigated for these four derivatives and compound **37** demonstrated promising leishmanicidal activity (Upadhyay et al., 2018).

Costa et al. synthesized and evaluated the anticancer and antileishmanial properties of several 4-substituted quinolones, compound **39** and **40** were reported to have the best anti-melanoma and anti-leukemia activity with the lowest EC_50_ values for decreased cell viability. Apoptosis dependent on caspases, the activity of cysteine protease, and mitochondrial permeabilization were reported to have been observed according to the mechanistic investigation. Another derivative, **41** had the best inhibitory activity against *L. mexicana* cysteine proteases type B. These findings suggest that 4-substituted quinolines could be useful as anticancer and antileishmanial drugs (Costa et al., 2020).

#### 3.1.5 α-glucosidase inhibitory activity

**FIGURE 9:**
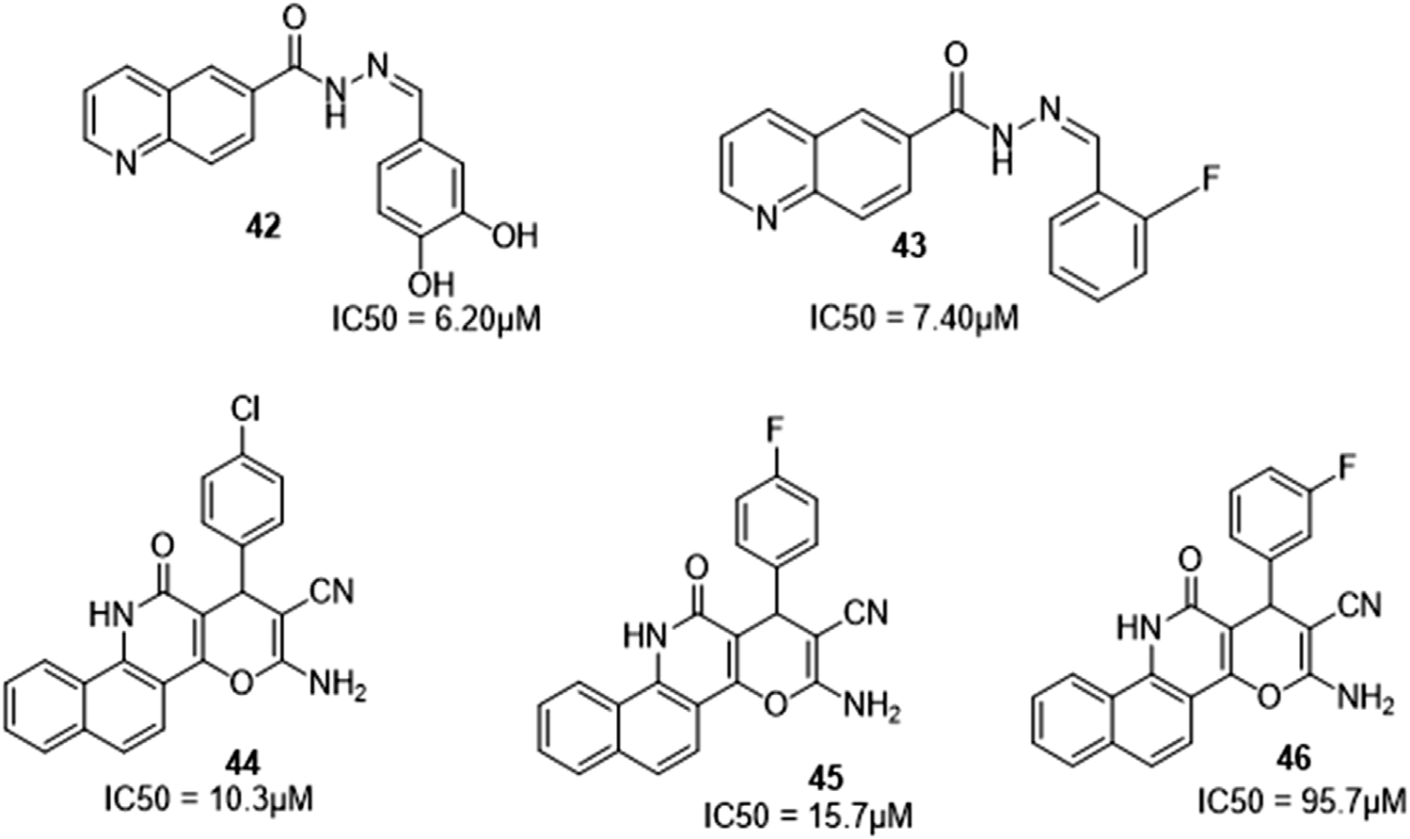
Quinoline hybrids with a-glucosidase inhibitory activity.

Urease, beta-glucuronidase, thymidine phosphorylase, and α-amylase are all inhibited by nitrogen-containing heterocyclic moieties. Taha et al. developed quinoline-Schiff base hybrids as a potent family of *in vitro* α-glucosidase (an enzyme that hydrolyzes polysaccharides and disaccharides, the main cause of diabetes mellitus) inhibitors **(** Figure 9 **)**. All derivatives inhibited α-glucosidase activity *in vitro* at doses ranging from 6.20 to 48.50 µM with acarbose as the reference drug. Derivatives with two OH groups, **42,** and a fluoro group, **43** were the most effective in the series (Taha et al., 2019).

Nikookar et al. synthesized dihydropyran-quinoline derivatives and investigated the *in vitro* inhibitory effects against α-glucosidase. The most potent derivatives were **44** (IC_50_ = 10.3 µM) with chloro substitution on position 4 on the phenyl ring and **45** (IC_50_ value of 15.7 µM) with fluoro substitution on position 4 on the phenyl ring. A modification of the fluoro group from position 4 to 3 on the phenyl ring lowered the inhibitory action as in **46** (IC_50_ = 95.7 µM) (Nikookar et al., 2018).

#### 3.1.6 Antioxidant activity

Investigation of the antioxidant capabilities of azoimine-quinoline hybrids by Douadi et al. was achieved by assessing the 1,1-diphenyl-2-picrylhydrazyl (DPPH) free radical scavenging activity of derivatives. The DPPH test was utilized to determine their reactivity. When the stable free radical DPPH is scavenged, it takes on a purple color and turns yellow (λ_max_ = 517 nm). Antioxidants react with DPPH to generate DPPH-H, which is a non-radical form. The concentration and absorbance at 517 nm of DPPH will decrease as a result. Various dosages of derivatives were used to assess a compound’s scavenging capacity of DPPH free radical. The results using these derivatives demonstrate that the DPPH radical’s absorbance reduced as the concentration was increased. At very low concentrations, the derivatives were reported to show greater antioxidant potential than ascorbic acid (Douadi et al., 2020).

**SCHEME 11:**
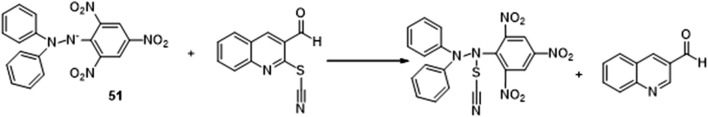
Mechanism of compound 51 scavenging activity.

The antioxidant activity of the quinoline derivatives synthesized by Zeleke et al. was determined using DPPH. All derivatives had moderate activity, with compound **51** having the highest activity (radical scavenging activity of 67% at a concentration of 100 g/mL). Compound **51** has no easily transferable hydrogen, it was proposed that the sulfur in the thiocyanate functional group donates an electron to the lone pair of the nitrogen atom’s electron, making a connection with sulfur as shown in Scheme 11 (Zeleke et al., 2020).

#### 3.1.7 Anti-inflammatory activity

**FIGURE 10:**
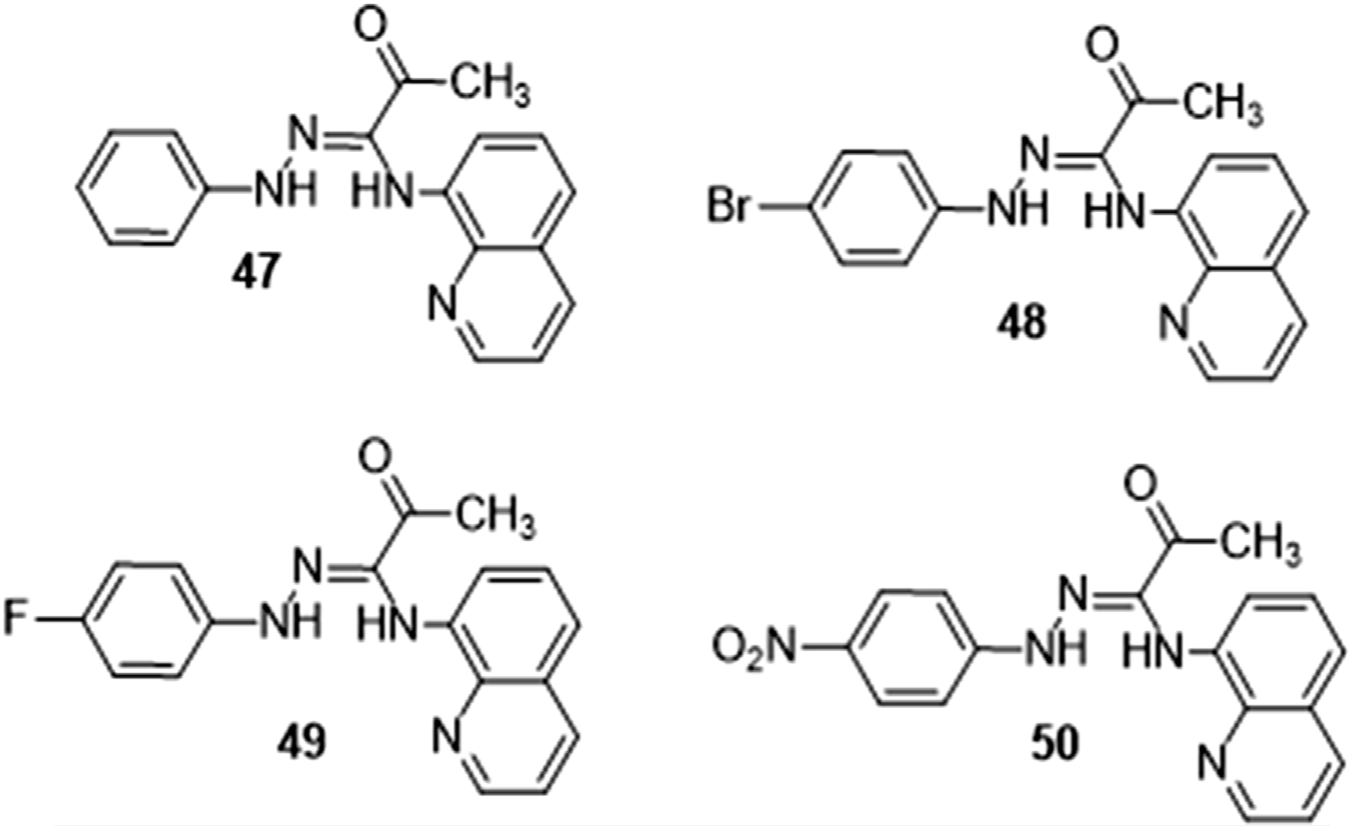
Derivatives with anti-inflammatory activity.

Douadi et al. investigated quinoline-azoimine hybrids’ anti-inflammatory capabilities *in vitro* by comparing their suppression of albumin denaturation to that of the gold standard, diclofenac sodium (Figure 10). Compounds **48** and **49** have anti-inflammatory characteristics similar to diclofenac, according to the research. Compounds **47** and **50** were found to decrease inflammation by 70.32% and 54.22% respectively at a concentration of 100 mg/mL. These figures are lower than those for diclofenac. Compound **49** showed the highest anti-inflammatory activity when compared to the others (Douadi et al., 2020).

**FIGURE 11:**
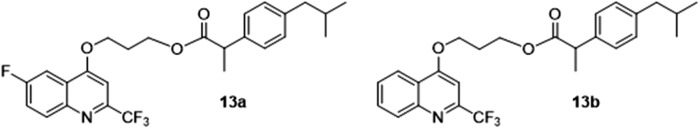
Ibuprofen-quinoline hybrids with anti-inflammatory activity.

Ghanim et al. evaluated ibuprofen-quinoline hybrids (Figure 11) and their anti-inflammatory properties to see if they were comparable to their progenitor (ibuprofen, a clinically authorized anti-inflammatory medicine that reveals its peak anti-inflammatory action at 3 h). It was reported that their anti-inflammatory effects were comparable to ibuprofen’s with the mean edema thickness of the control group staying nearly constant until it dropped at 24 h. The anti-inflammatory activity of most potent derivatives was low and mild at 1 h and 2 h respectively and then potency increased with time at 3 h and 4 h, indicating their promising activity at 3 h compared to the commonly used drugs (Ghanim et al., 2022).

#### 3.1.8 Antimalarial activity

**FIGURE 12:**
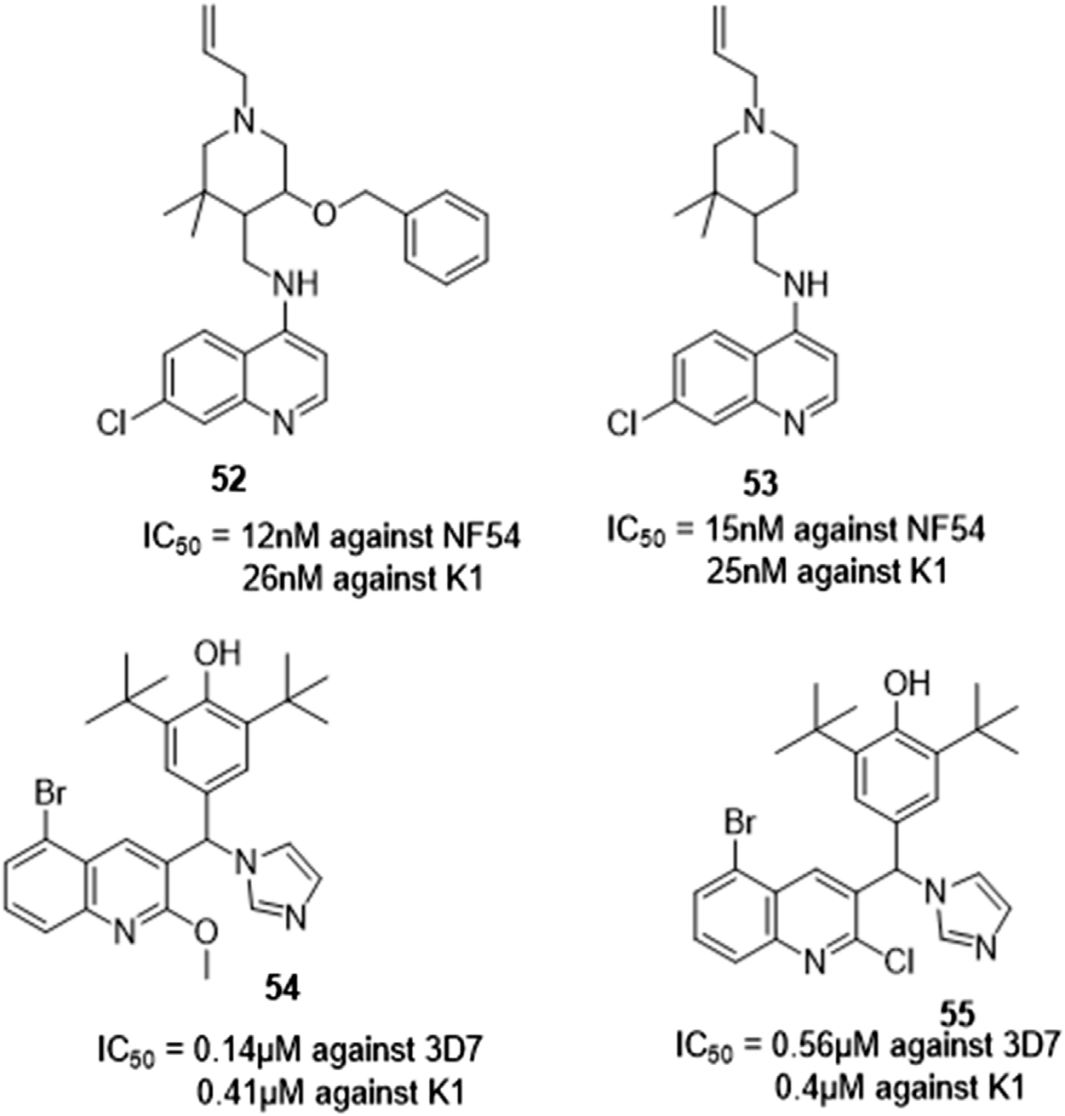
quinoline derivatives with antimalaria activity.

Van de Walle et al. synthesized quinoline-piperidine derivatives to test their antimalarial potential. Almost all derivatives showed promise against NF54 and K1 a chloroquine sensitive and a chloroquine-resistant *Plasmodium falciparum* strain respectively (Figure 12). *In vitro* evaluation of the compounds was reported to have demonstrated outstanding anti-plasmodium activity, with compounds **52** and **53** showing the greatest results with IC_50_ values of 12 nM and 15 nM against NF54 and 26 nM and 25 nM against K1 parasite strains respectively. Additionally, no cytotoxicity issues were discovered during biological testing in Chinese hamster ovary (CHO) cells. The derivatives were presented as promising structures for more effective antimalarial optimization treatments, given the critical need for novel antimalarial medications to fight the developing resistance of the parasite to artemisinins (Van de Walle et al., 2020).

Roy et al. synthesized quinoline imidazole hybrids and used an SYBR green-based fluorescence assay to assess antimalarial activity. Compounds **54** and **55** were reported to be the most active with IC_50_ values lower than the other derivatives. The activity of the other compounds were also reported to be moderately active against the CQ-sensitive strain falling within the range of 1.0–3.8 µM IC_50_ values.

Parasite inhibition as a function of stereochemistry was also investigated *via* enantiomeric separation of the racemic mixture of compounds. Asymmetric synthesis of one stereoisomer of compound **54** with negligible cytotoxicity and good selectivity index (SI) was investigated. A complete biological profile was recommended as well due to the reported observed increased activity when compared to the other stereoisomer. This will allow investigation of the biological activities of each enantiomer, assisting in the rationalization of the balance between antiparasitic activity, cytotoxicity, and resistance concerns (Roy et al., 2022).

### 3.2 Structural activity relationship (SAR) of some quinoline hybrids

**FIGURE 13:**
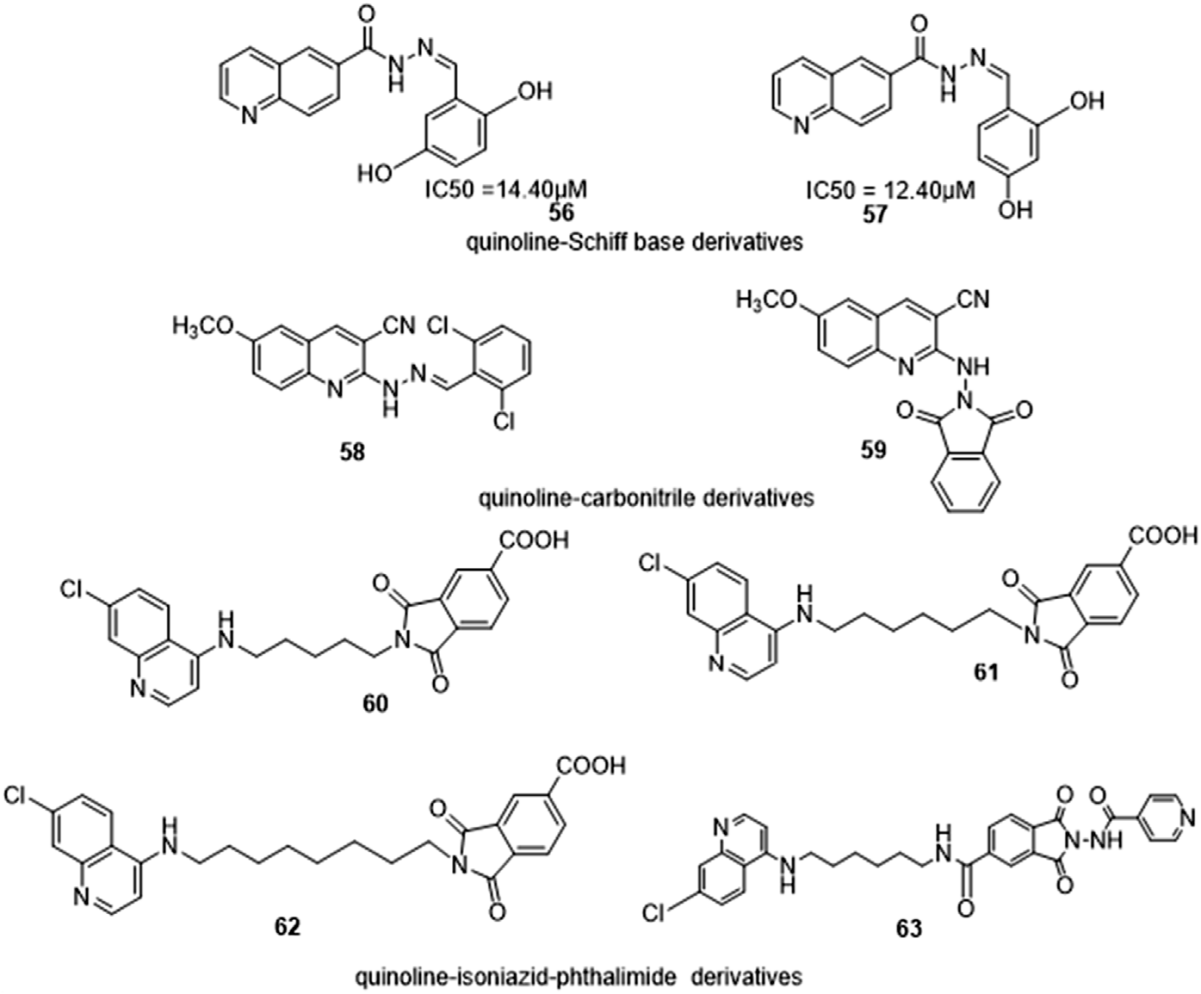
Some quinoline derivatives with reported SAR.

Quinoline-Schiff bases were synthesized by Almandil et al. and their inhibitory action was tested against α-glucosidase *in vitro* in the presence of the reference drug acarbose. It was reported that changing the position of hydroxyl on the phenyl ring reduced inhibitory activity, with derivatives containing two OH groups on the phenyl ring exhibiting excellent inhibitory activity (Figure 13). The SAR investigation showed that derivative **42**, a dihydroxy on positions 3 and 4 (IC_50_ = 6.20 µM) had the best anti-α-glucosidase activity. Derivatives **56** and **57** (IC_50_ = 12.40 and 14.10 µM) were discovered to have less inhibitory potential than **42** due to changes in the position of OH- groups from positions 3 and 4 (Almandil et al., 2019).

El-Gamal et al. synthesized quinoline-carbonitrile derivatives and found them to be promising antibacterial lead compounds by varying substitutions at position 6, indicating the biological preference of the methoxy group over methyl or without any substitution. Different substitutions on the quinoline molecule were investigated and SAR analyses of derivatives as possible antibacterial drugs. Compared to derivatives with methyl groups, it was reported that compounds **58** and **59** with methoxy group had increased inhibition zone (El-Gamal et al., 2018).

Rani et al. investigated the antiplasmodial activity of synthesized quinoline-isoniazid-phthalimide triads on *Plasmodium falciparum* CQR-W2 strain and accessed cytotoxicity on the Vero cell line of mammalian. The antiplasmodial and cytotoxic activities of synthesized triads were investigated with the reference drug being chloroquine (CQ). The antiplasmodial activity of the 4-aminoquinoline-phthalimides derivatives differs as the spacer length changes, as seen by IC_50_ values of 50.8, 30.2, and 43.2 nM for 60, 61, and 62 respectively. The triads were made by substituting the quinolone with isoniazid (INH) around the phthalimide core giving rise to the most promising derivative in series **63** with an IC_50_ value of 11 nM (Rani et al., 2021).

**FIGURE 14:**
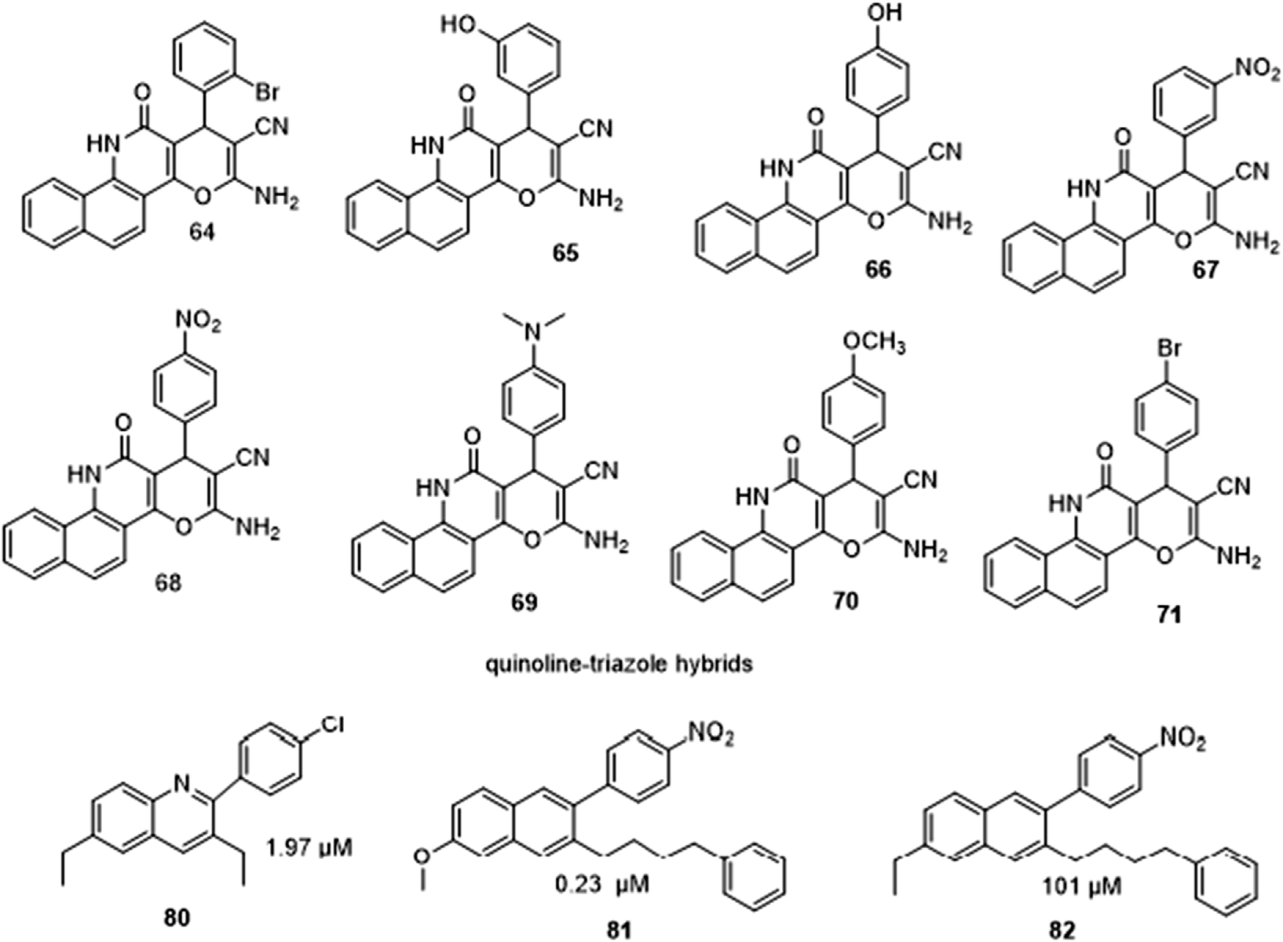
SAR studies showing change in potency of quinoline derivatives as substituent changes.

Ramprasad et al. synthesized target quinoline-triazole hybrids (Figure 14) and found that the hybrids with fluoro on position 3 of the triazole, **16** and **17** with the n-octyl group were reported to be the most promising leads. The type of substituents on the 1,2,3-triazole affected the compounds’ activity. Meta substitution of Fluorine on the phenyl ring increased activity, however, fluorine on ortho- and para-position of the phenyl did not. When IC_50_ values of **66** and **68** which are 4-substituted derivatives were compared to **65** and **67** the 3-substituted derivatives the results demonstrated that the 4-position is more effective. Furthermore, the 4-Bromo derivative 51 outperformed the 2-Bromo derivative **64** in terms of activity. This revealed that the activity is influenced by the character of substituent on the 4-position of the phenyl. Furthermore, compounds **69** and **70** were reported to inhibit a-glucosidase in virtually identical ways even though their substituents on the 4-position of the phenyl ring differ in electron affinity; hence, in these groups of compounds, the inhibitory action was dependent on the substituent position (Ramprasad et al., 2019).

**FIGURE 15:**
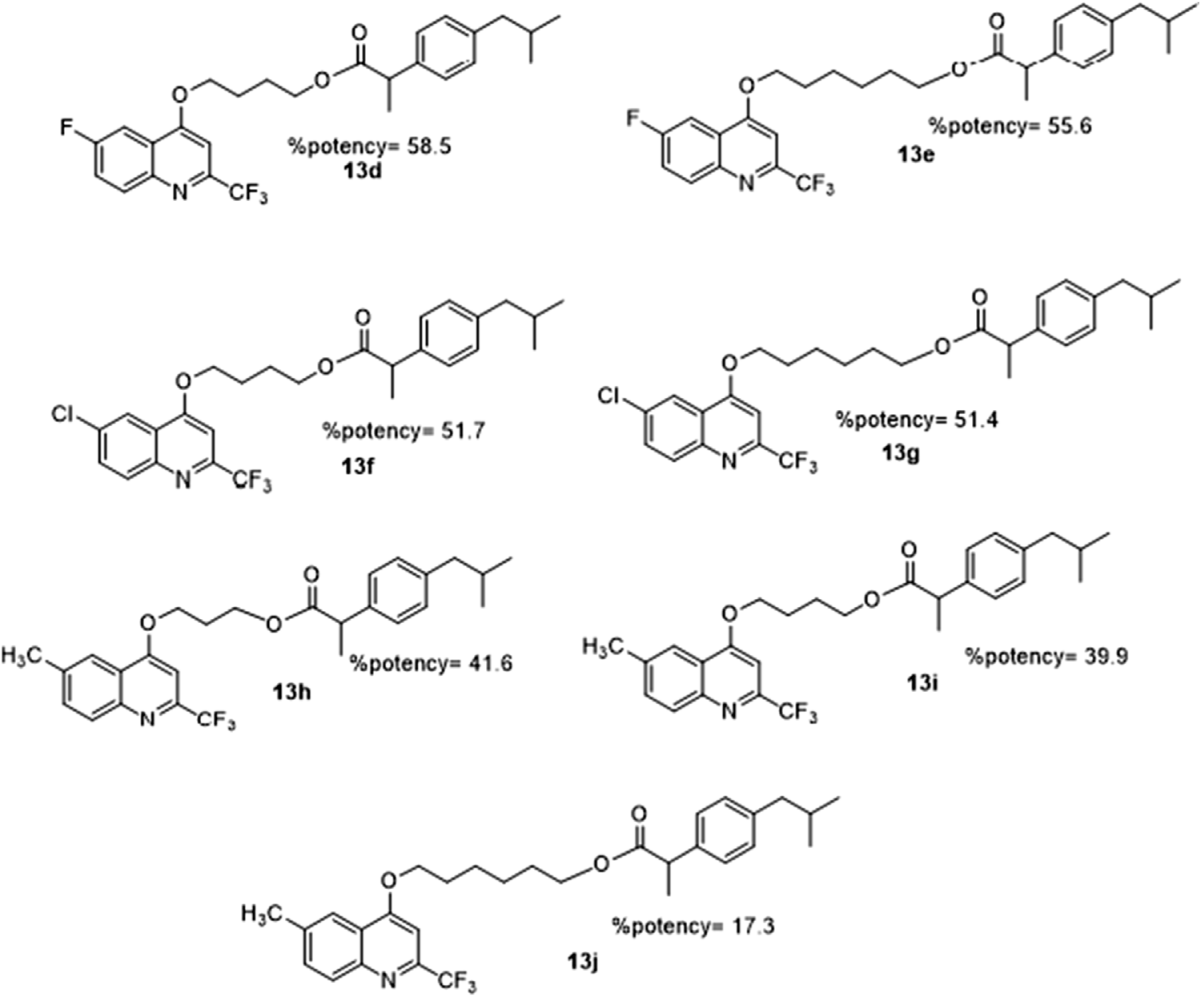
SAR activity of Ibuprofen-quinolinyl hybrids.

Ghanim et al. observed SAR in manufactured ibuprofen-quinolinyl hybrids and reported that the bio-properties exhibited are dependent on the substituent of the quinolinyl heterocycle as shown in (Figure 15). Compounds **13c**, **13f,** and **13g** (the Cl substituted derivatives), **13b**, **13d,** and **13e** (the F substituted derivatives), and **13h, 13i,** and **13j** (methyl-substituted) show that the fluorine substituent out-performs chlorine and methyl in terms of percent potency. The alkyl chain that connects ibuprofen to the quinolinyl heterocycle influences bioactivity as its length differs. The substituent with 3 carbon alkyl linker was reported to be more suited for exhibiting higher anti-inflammatory activity than those with 4 and 6-carbon (Ghanim et al., 2022).

**FIGURE 16:**
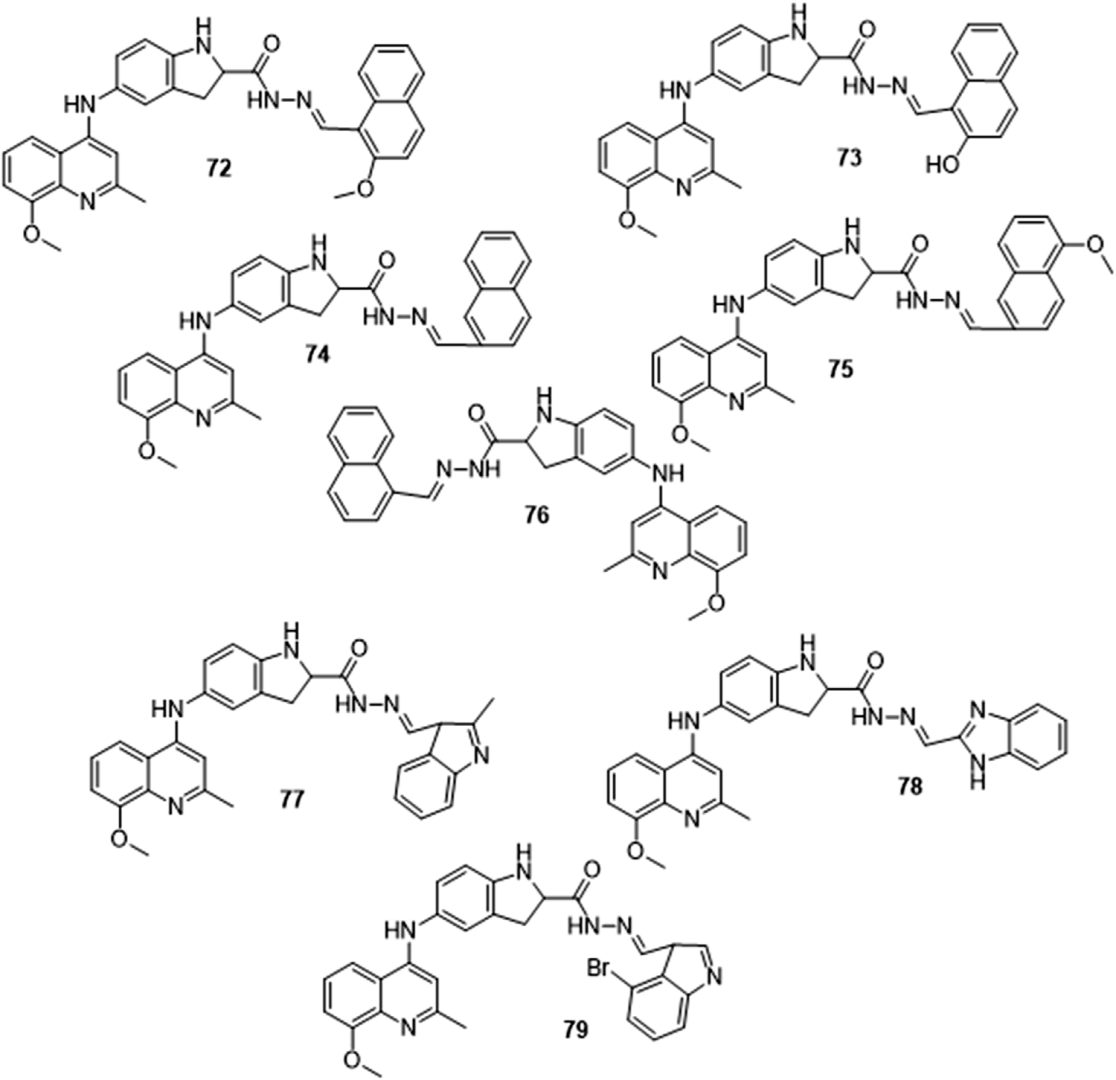
Quinoline-Indole-Schiff base derivative.

The Quinoline-Indole-Schiff base derivative synthesized by B. Li et al. was reported to have excellent antiproliferative activities and did not exhibit any cell toxicity in all the tested tumor cell lines (Figure 16). IC50 values of derivatives **71**, **72, 73, 74, 75,** and **76** range from 1.24 to 4.95 µM and showed a π-π interaction between Nur77-LBD and the naphthalene ring. The substitution of naphthalene at the *N′*-methylene site with indole, as in **77,** and other bicyclic aromatic rings as in **78** and **79** reduced antiproliferative activity with IC_50_ values greater than 20 μM, indicating that the bicyclic ring attached to N′-methylene affects activity (Li et al., 2021).

Hayat et al. synthesized a variety of substituted quinoline derivatives and tested their inhibitory activity against h-NTPDase, the derivatives were reported to inhibit h-NTPDase1 to varying degrees, with IC_50_ ranging from .23 to 101.0 µM. The standard used was Suramin with an IC50 of 16.1 µM. Derivatives with an alkyl or benzyloxy propyl group in the quinoline ring were reported to be the most potent with IC_50_ values ranging from 1.97 to 19.3 µM. Inhibitory activity of derivatives with an alkyl group was reported to be good to moderate whereas compound **80** with a simple methyl group had an IC_50_ value of 1.97 µM and showed strong inhibition of h-NTPDase1. Compound **81** having a NO_2_ group and an OCH_3_ group with an IC_50_ value of .23 µM was reported to be the most effective inhibitor of h-NTPDase1 in this series. Although the methoxy group is an electron donor, however in this compound is reported to withdraw electrons inductively, hence the stronger inhibitory effect was attributed to the combination of two groups withdrawing electrons, as the NO_2_ group is also an electron-withdrawing group. The decrease in h-NTPDase1 inhibition when the OCH_3_ group was substituted with an electron-giving substituent like CH_3_ in **82** led to this conclusion (Hayat et al., 2019).

### 3.3 ADMET prediction of some selected derivatives

ADMET properties combine drug pharmacokinetic properties including absorption, distribution, metabolism and excretion, and drug pharmacodynamics properties including drug efficacy and toxicity. These properties help in optimization and facilitate the selection of drug candidates with the best safety and pharmacological profile while understanding the mechanisms behind their activity. They influence the bioavailability of drugs orally, absorption in the bio-membrane, and metabolism (Kalita et al., 2019).

**FIGURE 17:**
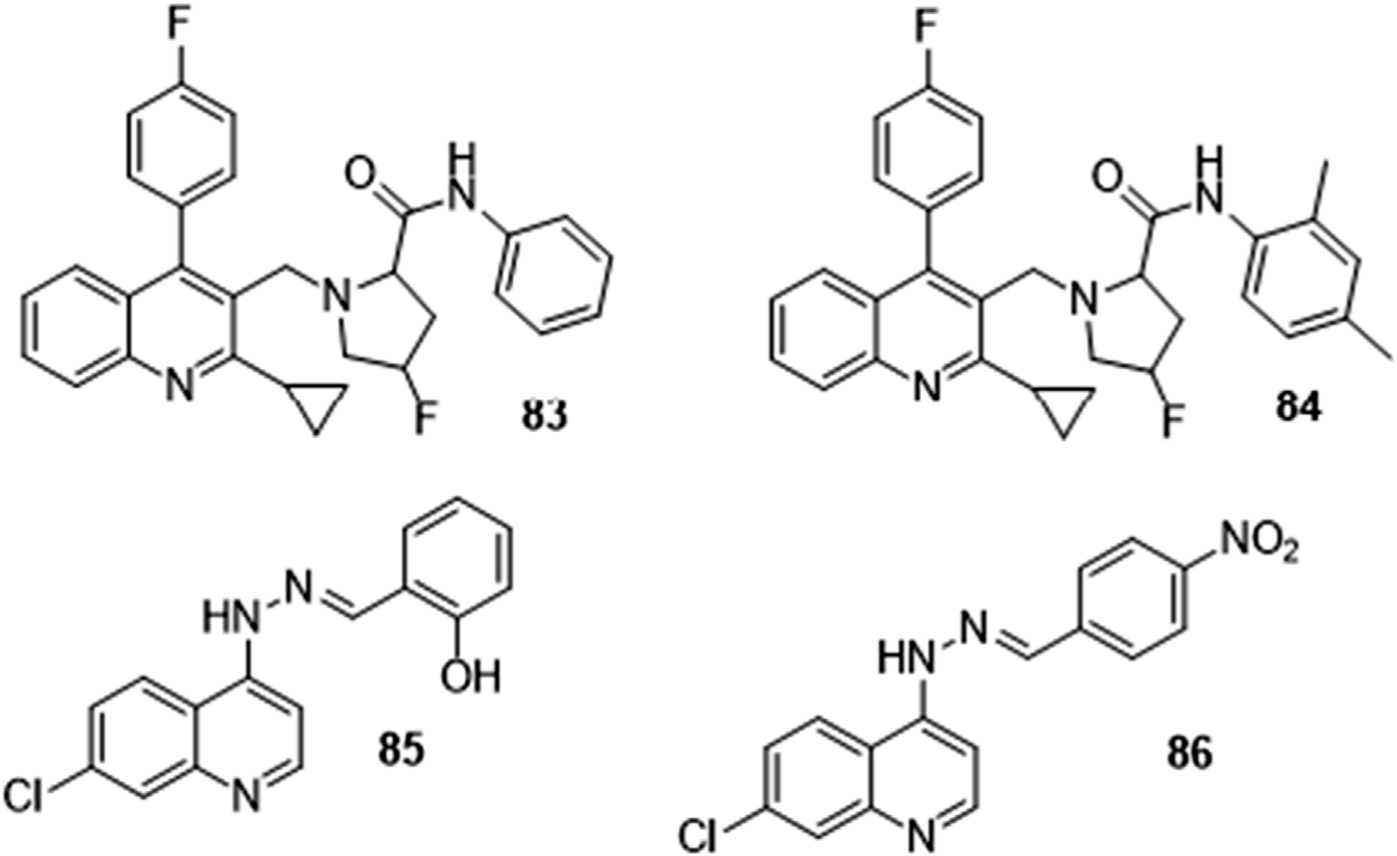
Quinoline-fluoroproline amide and quinoline-imine hybrids with reported insilico ADMET.

Various quinoline derivatives (Figure 17) have been reported with their ADMET properties indicating their drug-likeness, some of which are stated below.

Ganesan et al. synthesized a series of quinoline-fluoroproline amide hybrids including **83** and **84** and predicted their ADMET parameter *insilico* and stated that the derivatives obeyed the Lipinski rule of five and exhibited low skin permeability. They were reported to inhibit P-Glycoprotein I and II with a very low volume of distribution, Central Nervous System (CNS), and Blood Brain Barrier (BBB) permeability. The hybrids were also reported to be CYP2D6, and CYP3A4 inhibitors and have no inhibitory activity against the CYP1A2, CYP2C9, and CYP2C19 enzymes of the cytochrome P450 which many drugs rely on for metabolism. Toxicity tests of anticipated chemicals proved that they were non-toxic and non-Mutagenic (Ganesan et al., 2020).

Kalita et al. carried out an *in silico* ADMET study on a series of quinoline-imine derivatives and reported that derivatives had excellent intestinal absorption and are soluble in water, they were non-inhibitors of cytochrome CYP2D6 and had mild to moderate BBB penetration (as they are intended for use in the treatment of cerebral malaria). They also differed in the level to which they bound with protein. Compounds **85** and **86** were reported to be the most potent of this series of derivatives (Kalita et al., 2019).

## 4 Conclusion and recommendation

Quinoline derivatives including their hybrids are important structural scaffolds in therapeutically effective molecules in medicinal chemistry research. Ther pharmacological activities necessitate a review of its chemistry and biodiversity hence their biological applications including their excellent paths to bind to biomolecular targets have been examined in this study. This further confirms their usefulness as drug design precursors and for the development of novel therapeutic prospects. Identification of moieties that have the potential for improved activities when coupled with quinoline to form hybrids is important. In synthesizing derivatives that are therapeutic candidates for the treatment of diverse infectious diseases. These hybrids can have better selectivity and dual mode of action in a bid to overcome drug resistance. They can then be characterized, validated, optimized, screened, and tested to confirm treatment efficacy. Once proven to be of use, the drug development process before clinical trials can begin.
